# Measuring body checking and avoidance in adolescents: validation of a behavioral questionnaire

**DOI:** 10.1186/s40359-026-05614-y

**Published:** 2026-09-18

**Authors:** Clara Sophie Beer, Stephan Zillmer, Nelly Drießnack, Charlotte Jaite, Tanja Legenbauer

**Affiliations:** 1 LWL University Hospital Hamm for Child and Adolescent Psychiatry, Psychotherapy and Psychosomatic, Ruhr-University Bochum, Hamm, Germany; 2 Institute of Psychology, Clinical Psychology and Psychotherapy of Childhood and Adolescence, University of Hildesheim, Hildesheim, Germany; 3 Department of Child and Adolescent Psychiatry, Psychosomatic Medicine and Psychotherapy, Charité-Universitaetsmedizin Berlin, corporate member of Freie Universitaet Berlin, Humboldt Universitaet zu Berlin, Berlin Institute of Health, Berlin, Germany

**Keywords:** Eating disorders, Body image, Body checking, Body avoidance, Adolescence

## Abstract

**Background:**

Body image distortion (BID) is central to eating disorder (ED) pathology and is closely linked to body dissatisfaction. While most questionnaires assess affective, cognitive, or perceptual components of body image, the behavioral component manifesting in body checking (BC) and body avoidance (BA) has received comparatively little attention. Given that EDs typically emerge during adolescence, validated measures of behavioral BID are particularly needed for this age group. The Body Checking and Avoidance Questionnaire (BCAQ) is the only instrument assessing both BC and BA, but it has not yet been validated in adolescents.

**Methods:**

The present study aimed to evaluate the BCAQ in a clinical sample of female German adolescents with (atypical) anorexia nervosa. Factor structure, internal consistency, and convergent and known-groups validity were examined.

**Results:**

An initial confirmatory factor analysis of the original 27-item BCAQ indicated poor model fit. An exploratory factor analysis identified four items with low or inconsistent loadings. Analyses of the resulting 23-item version yielded a preliminary three-factor structure and demonstrated good reliability (Cronbach’s α = 0.78–0.94) and satisfactory convergent validity, while known-groups comparisons provided limited and exploratory evidence, with a small group difference observed only for avoidance behavior.

**Conclusion:**

These findings provide preliminary evidence for the use of the adapted 23-item BCAQ to assess behavioral aspects of body image distortion in adolescents with (atypical) anorexia nervosa. Further longitudinal research is needed to evaluate the measure’s temporal stability.

**Supplementary Information:**

The online version contains supplementary material available at https://doi.org/10.1186/s40359-026-05614-y.

## Abstract (plain language summary)

This study examined whether the Body Checking and Avoidance Questionnaire can be used reliably with adolescents. Body image refers to how people think and feel about their own body and how they behave as a result. For some individuals, this includes behaviors such as frequently checking their body or avoiding looking at it. These behaviors are common in people with eating disorders. Eating disorders often begin during adolescence, but many questionnaires have mainly been developed and tested in adults. This makes it unclear whether they are suitable for younger people. In this study, we tested the questionnaire in female adolescents with an anorexia nervosa or atypical anorexia nervosa and compared their results with those of adults. Our findings suggest that the adapted questionnaire is a promising tool for assessing body checking and body avoidance in female adolescents with anorexia nervosa. Further research is needed to examine how well the questionnaire performs over time and in other groups of adolescents before it can be recommended for broader clinical use.

**Supplementary Information:**

The online version contains supplementary material available at https://doi.org/10.1186/s40359-026-05614-y.

## Background

In recent decades, body image has received considerable attention in research involving both clinical and non-clinical populations [1–3]. Body image is a multidimensional construct encompassing perceptual, affective-emotional, cognitive, and behavioral components, including behaviors such as body checking or body avoidance [4–6]. Adolescence is a particularly vulnerable period in the development of body image, due to the onset of puberty and changing social dynamics. During this phase, body-related concerns often increase [7], which may contribute to the development of body dissatisfaction and, subsequently, to body image distortion (BID) [8]. Girls appear to be particularly vulnerable, with nearly every second girl affected by BID [9–13]. Furthermore, higher body dissatisfaction and dieting behaviors tend to increase with age [14, 15]. These patterns are particularly concerning given that such factors are well-established risk contributors to the development of eating disorders (EDs). EDs involve persistent disturbances in eating behaviors and related thoughts or emotions, often centered on weight, body shape, and food. Given the elevated risk of developing an ED [16], the persistence of BID into adulthood [17, 18] and its association with depression [19], it is crucial to identify early signs of body image dissatisfaction or distortion.

Two behavioral components of body image distortion, body checking (BC) and body avoidance (BA), are regarded as key maintaining factors in EDs [20]. BC is defined as the repetitive inspection of one’s body, particularly of body areas perceived as flawed. In contrast, BA describes efforts to avoid body-related stimuli or situations, such as covering mirrors or wearing loose clothing to hide body shape [21]. Although numerous questionnaires assess body image on the affective-emotional, cognitive, or perceptual level (e.g. [22], [23], [24], few instruments exist that specifically address the behavioral level. To our knowledge, only five questionnaires measure BC and BA: the Body Shape Questionnaire (BSQ; [25]), the Body Image Avoidance Questionnaire (BIAQ; [26]), the Body Image Concern Inventory (BICI; [27]), the Body Checking Questionnaire (BCQ; [28]), and the Body Checking and Avoidance Questionnaire (BCAQ; [21]). The BSQ assesses eating attitudes, body dissatisfaction and includes four single items assessing BA and BC [25]. The questionnaire itself demonstrates excellent test–retest reliability (*r* = .88), satisfactory concurrent validity [29] and high internal consistency (e.g., Pook et al., [30]. One validation study was conducted with adolescents [31], however, BA and BC were not evaluated using a separate scale. Similarly, the BICI includes single items for BA and BC. The questionnaire demonstrates high internal consistency (α = 0.93) and good concurrent and convergent validity [27], but lacks validation for adolescent populations and the behavioral components. The BIAQ, which focuses on BA, shows satisfactory psychometric properties with Cronbach’s alpha of 0.89 and test–retest reliability of 0.87 [32]. It has been adapted into various languages [33–36], including a version for German adolescents [32]. However, it does not assess BC. The BCQ, in contrast, was developed specifically to measure BC and comprises 23 items. It demonstrates acceptable internal consistency and excellent reliability of 0.94 [37]. The BCAQ is the only available instrument that captures both BC and BA. The items were developed through interviews with experts in BC and BA and were subsequently evaluated by patients with EDs [33]. However, to date, there is no validated adolescent version. As adolescents are in a period of ongoing physical, psychological and psychosexual development, some items referring to intimacy and sexuality may not be equally applicable across all age groups. Therefore, the present study aimed to investigate the factor structure of the BCAQ and assess its applicability in adolescent populations.

## Methods

### Participants

#### Sample 1

The examination of the three-factor structure, reliability, and convergent validity of the BCAQ was carried out using a sample of female adolescent inpatients with AN (*n* = 248) who had been assessed between 2019 and 2024 at the LWL University Hospital Hamm, Ruhr University Bochum as part of an ED-specific diagnostic and treatment program. The study was approved by the Ethics Committee of the Faculty of Medicine, Ruhr University Bochum (registration no. 4359-12), which waived the requirement for informed consent to participate for analyses of anonymized routine clinical data in accordance with Article 9(2)(j) of the GDPR and §§ 5(5), 6, and 17 of the DSG NRW. Patients and their parents or legal guardians were informed in writing that anonymized data might be used for research purposes. This information procedure was reviewed and approved by the ethics committee. For participants younger than 16 years of age, the requirement for parental consent was also waived under the same ethics committee approval due to the use of anonymized routine clinical data. Patients completed the diagnostics within the first 14 days after hospital admission under the supervision of trained psychologists or psychotherapists. Inclusion criteria were female sex, age younger than 18 years, and a primary or secondary ICD-10 diagnosis (Dilling et al. 2015): F50.0 (anorexia nervosa (AN)), F50.00 (AN, restricting type), F50.01 (AN, active type), or F50.1 (atypical AN). Exclusion criteria were the comorbid presence of any other ED diagnosis. After applying the inclusion and exclusion criteria, the final sample comprised *n* = 147 (sample 1; see Table 1a). Sample 1 was used for all psychometric analyses of the adolescent BCAQ, including the CFA, EFA, internal consistency, and convergent validity analyses.

**Table 1:** Characteristics of sample 1 and 2 divided by age groups

	Sample 1 (*n* = 147)	Sample 2 (*n* = 202)	
		Subsample A (*n* = 134)	Subsample B (*n* = 68)
	AN-Adolescents	AN-Adolescents	AN-Adults
**Demographics**			
Age range (years)	11–17	11–17	18–57
Age, *M* (*SD*)	15.03 (1.53)	15.00 (1.56)	26.5 (9.42)
BMI (kg/m^2^), *M* (*SD*)	16.23 (2.32)	15.96 (2.05)	16.71 (1.77)
BMI-percentile, *M* (*SD*)	11.2 (19.1)	9.26 (16.66)	-
**Clinical Characteristics**			
AN (main or secondary diagnosis), *n* (*%*)	147 (100%)	-	-
Main diagnosis, *n* (*%*)	131 (89.1%)	-	-
F50.0	21 (14.3%)	-	-
F50.00	64 (43.5%)	-	-
F50.01	27 (18.4%)	-	-
F50.1	19 (12.9%)	-	-
Secondary diagnosis, *n* (*%*)	16 (10.9%)	-	-
F50.01	2 (12.5%)	-	-
F50.1	14 (87.5%)	-	-
Any comorbidity, *n* (*%*)	105 (71.4%)	-	-
F10-19	1 (1.0%)	-	-
F20-29	1 (1.0%)	-	-
F30-39	72 (68.6%)	-	-
F40-49	22 (21.0%)	-	-
F60-69	2 (1.9%)	-	-
F80-89	3 (2.9%)	-	-
F90-99	28 (26.7%)	-	-
Inpatient treatment duration (days), *M* (*SD*)	90.07 (44.4)	-	-
**BCAQ (Original**,** 27 items)**			
Checking behavior, *M* (*SD*)	2.69 (0.88)	2.71 (0.87)	2.9 (0.66)
Avoidance behavior, *M* (*SD*)	2.24 (0.66)	2.23 (0.67)	2.6 (0.67)
Reassurance seeking, *M* (*SD*)	1.96 (0.91)	1.89 (0.88)	2.01 (0.91)
Total score, *M* (*SD*)	2.41 (0.68)	2.41 (0.68)	2.67 (0.55)
**BCAQ (Adapted**,** 23 items)**			
Checking behavior, *M* (*SD*)	2.69 (0.88)	2.71 (0.87)	2.9 (0.66)
Avoidance behavior, *M* (*SD*)	2.33 (0.78)	2.33 (0.80)	2.6 (0.73)
Reassurance seeking, *M* (*SD*)	1.96 (0.91)	1.89 (0.88)	2.01 (0.91)
Total score, *M* (*SD*)	2.47 (0.73)	2.47 (0.73)	2.68 (0.57)
**CDRS**			
Difference score felt – ideal, *M* (*SD*)	3.04 (2.54)	-	-
Difference score actual – ideal, *M* (*SD*)	1.27 (1.97)	-	-
**EDE-Q**			
Restraint, *M* (*SD*)	3.41 (1.94)	3.43 (1.92)	4.52 (1.39)
Eating concern, *M* (*SD*)	3.04 (1.49)	3.08 (1.49)	4.1 (1.23)
Weight concern, *M* (*SD*)	3.65 (1.74)	3.69 (1.7)	5.25 (1.26)
Shape concern, *M* (*SD*)	4.17 (1.76)	4.19 (1.73)	5.41 (1.14)
Total score, *M* (*SD*)	3.57 (1.59)	3.6 (1.56)	4.82 (1.1)

Note. Data for categorical variables are presented as counts and percentages (%), and data for continuous variables as means and standard deviations (SD). AN = anorexia nervosa. Case definition of AN was according to ICD categories F50.00, F50.0, F50.01, and F50.1. In Sample 1, cases with AN as either a primary or secondary diagnosis were included, provided that no comorbid eating disorder was present. In Subsample A, only cases with AN as the primary diagnosis were included

#### Sample 2

For the comparison between adolescents and adults (known-groups validity), a second analytical sample (Sample 2) was created. This sample consisted of two subsamples. An adolescent subsample derived from the same source population as Sample 1 and an adult subsample obtained from the original BCAQ validation study: The first subsample (Subsample A) of female adolescents derived from the same source population (*n* = 248) from which Sample 1 was also formed. Due to comparability with the adult subsample, only cases with a primary diagnosis (not secondary diagnoses) in the aforementioned ICD-10 categories (F50.0, F50.00, F50.01, F50.1) were included. Otherwise, the same inclusion and exclusion criteria applied for Sample 1 (with regard to sex, age, and other comorbid EDs). After applying the inclusion and exclusion criteria, this subsample resulted in *n* = 131. The second subsample of adult women (subsample B) comprised participants from two previously reported datasets (see [33]): 56 patients with a primary ED diagnosis recruited from the University of Mainz outpatient clinic and 385 individuals recruited online (*n* = 422). Inclusion criteria were female sex, an age of 18 years or older, and the presence of AN (only primary diagnoses were available for this data set). After applying the inclusion and exclusion criteria, the final adult subsample comprised *n* = 68 participants. For the adult comparison sample, the same four items omitted in the adolescent sample were excluded from the adult dataset. Subscale and total mean scores were then calculated using the same scoring procedure as for the adapted 23-item BCAQ. Three patients were younger than 18 years of age and were therefore reassigned to Subsample A. Based on the composition of the adolescent subsample (subsample A; *n* = 134) and the adult subsample (subsample B; *n* = 68), sample 2 thus consisted of *n* = 202 (sample 2; see Table 1a).

### Measures

#### Body checking and avoidance questionnaire (BCAQ)

The German version of the 27‑item Body Checking and Avoidance Questionnaire (BCAQ), validated for eating‑disorder populations [33], was used to assess the dimensions of “checking behavior”, “avoidance behavior”, and “reassurance seeking”, and to examine whether this factor structure could be replicated in a sample of adolescents with AN. Items are rated on a four‑point Likert scale ranging from 1 (“not at all”) to 4 (“very much”), indicating how well each description reflects the respondent’s behavior. Scoring was performed by calculating mean scores for each subscale and a total score as the mean of all items.

#### Eating disorder examination-questionnaire (EDE-Q)

The Eating Disorder Examination Questionnaire (EDE-Q; [38]; German version: [39]) was used to assess ED psychopathology on the dimensions of “restraint”, “eating concern”, “weight concern”, and “shape concern” within the last 28 days. Items are answered on a scale from 0 (“never”) to 6 (“always”), categorically between 0 (“no”) and 1 (“yes”), or in an open format. The German version of the EDE-Q has demonstrated good convergent and known-groups validity as well as (retest) reliability [40]. To assess the convergent validity of the BCAQ, particular emphasis was placed on the subscales “weight concern” and “shape concern”, as these relate to body-related control and avoidance behavior and are most closely linked to the BCAQ construct. As with the BCAQ scoring, mean scores were calculated for each subscale in addition to a total score.

#### Contour drawing rating scale (CDRS)

Data from the Contour Drawing Rating Scale (CDRS; [41]) were also used to evaluate convergent validity. The CDRS consists of a range of standardized body silhouettes that gradually increase in weight from 1 (body silhouette with the least weight) to 8 (body silhouette with the highest weight) [42]. Participants use these silhouettes to answer the following questions: “What do you actually look like?” (“actual body silhouette”), “How do you feel?” (“felt body silhouette”) and “How would you like to look?” (“ideal body silhouette”) [43]. Based on these responses, difference scores are calculated that imply the discrepancy between the felt body and the ideal body (“felt body” - “ideal body”) and between the actual body and the ideal body (“actual body” – “ideal body”) [44]. The discrepancy between felt and ideal body silhouette is strongly correlated (*r* = .58, *p* < .001) with indicators of body dissatisfaction measured using the “shape concern” scale of the EDE-Q [38, 45]. The CDRS has a high internal consistency of Cronbach’s 𝛼 = 0.78 [33, 41] and high construct validity [46]. To evaluate the convergent validity of the BCAQ, the discrepancy score between the felt and ideal body silhouette was used.

### Statistical analyses

Mean values, medians, and standard deviations were calculated for the individual items and the subscales of the BCAQ. The distributions of the respective samples were assessed for normal distribution and the appropriate statistical analysis was selected. For comparison, the mean values and standard deviations of the CDRS and EDE-Q scales are also presented (see Table 1a).

### Factor analysis and investigation of the factor structure

For the psychometric evaluation of the BCAQ in an adolescent sample, the factor structure was examined using confirmatory factor analysis (CFA) for the three-factor structure validated in adults [33]; “checking behavior”, “avoidance behavior”, “reassurance seeking”. Following this three-factor structure, the factor “checking behavior” contains 12 items, the factor “avoidance behavior” contains 12 items, and the factor “reassurance seeking” contains 3 items. All 27 items and all participants in Sample 1 were included in the CFA analysis. In addition, all factors were standardized [47] and, due to non-multivariate normally distributed variables (Mardia skewness = 4921.19, *p* < .001; Mardia kurtosis = 9.65, *p* < .001), the robust maximum likelihood method [48] was used as an estimator. Missing data were handled using the full information maximum likelihood (FIML) approach under the assumption of missing at random (MAR), as this method provides unbiased and more efficient estimates using all available data [49]. Given the low overall percentage of missing data (0.8%) and the absence of a systematic missing data pattern, the data were considered to meet the assumption of MAR necessary for FIML estimation. The adequacy of the final sample size of sample 1 used for CFA (*n* = 147) was evaluated based on [50], who showed that required sample sizes depend on the number of indicators and factors, and the magnitude of factor loadings. For the third factor “reassurance seeking”, which comprises only three indicators, Wolf et al., [50] indicated a minimum sample size of approximately *n* = 150, given high loadings (*λ* ≥ 0.80) observed in the adult validation sample [33]. For the remaining two factors (“checking behavior” and “avoidance behavior”; 12 indicators each), the required sample size is lower (approximately *n* = 100 for *λ* ≥ 0.65). Even assuming a more conservative estimate with moderate loadings (*λ* ≥ 0.50), the recommended sample size remains approximately *n* = 150. While a larger sample size would have been preferable (e.g., *n* > 200; [51], the available sample of 147 was deemed adequate to reliably estimate the model parameters. To check the adequacy of the data, Bartlett’s test of sphericity and the Kaiser-Meyer-Olkin measure of sampling adequacy were applied. To check the model fit, a goodness-of-fit analysis was performed using various (robust) indices (SRMR, RMSEA, RCFI, RTLI; [52]). Following commonly used recommendations, RMSEA values below 0.08, SRMR values below 0.08, and CFI and TLI values of at least 0.90 were considered indicative of acceptable model fit, whereas χ²/df values below 3 were considered acceptable [47, 53]. Due to the aforementioned violations of normal distribution, Spearman’s correlation coefficients were calculated for the intercorrelations between the factors. The CFA was performed in RStudio (version: 2024.12.1 + 563), using the packages lavaan [54] and foreign (R Core [55]).

The primary aim of the factor-analytic approach was to examine whether the original three-factor structure of the BCAQ, previously established in an adult ED sample [33], could be replicated in an adolescent clinical sample. Therefore, a CFA was conducted first. As the CFA indicated poor model fit, an exploratory factor analysis (EFA) was subsequently performed to explore the underlying factor structure of the BCAQ in adolescents and to identify potentially problematic items. Accordingly, no modifications to the original model based on modification indices were performed, as the aim was to explore the factor structure in this adolescent clinical sample. For the EFA, missing data were handled by excluding cases pairwise.

### Reliability analysis

The internal consistency of the three subscales was examined using a reliability analysis, and the respective Cronbach’s 𝛼 coefficients were calculated. Changes in Cronbach’s 𝛼 coefficients were examined, taking into account the removal of certain items (based on factor analysis). In addition, the item difficulties and Spearman inter-scale correlations were calculated. For reliability analyses, listwise exclusion was applied.

### Convergent and known-groups validity

To evaluate the convergent validity of the BCAQ, Spearman correlations were calculated for the three subscales of the BCAQ with corresponding scales and questionnaires (CDRS [41] and EDE-Q [39] for assessing ED psychopathology in adolescents. Due to the highest construct similarity to the BCAQ, the evaluation of convergent validity was primarily based on the difference score “felt body - ideal body” of the CDRS [41] and the subscales “weight concern” and “shape concern” of the EDE-Q [38].

To check the known-groups validity, the three BCAQ subscales and the BCAQ total score were compared between a sample of adolescents and a sample of adults (see also Sample 2; Table 1a). As significant deviations from normality (*p* < .05) were found in Shapiro-Wilk tests for all subscales and the total score of the BCAQ, a non-parametric procedure (Mann-Whitney U test) was used. All statistical analyses (except the CFA) were performed in JASP [56] and IBM SPSS Statistics (IBM Corp. 2019). For correlation analyses and group comparisons, missing data were handled by excluding cases pairwise.

## Results

### Sample characteristics

For an overview of the samples 1 and 2 (and subsamples) see Table 1.

To check the comparability between the adolescent group (subsample A) and the adult group (subsample B), Mann-Whitney U tests were performed for differences in the EDE-Q scale (see Table 2).

**Table 2:** Mann-Whitney *U* tests of EDE-Q between subgroups (adolescents vs. adults) of sample 2 (*n* = 202)

		*Median*	*U*	*Z*	*p*	Cohen’s *r*
EDE-Q						
Restraint	Subsample A	4.00	5949.000	3.85	< 0.001	0.28
	Subsample B	5.00				
Eating concern	Subsample A	3.20	6304.000	4.57	< 0.001	0.32
	Subsample B	4.10				
Weight concern	Subsample A	4.20	7039.500	6.46	< 0.001	0.46
	Subsample B	5.60				
Shape concern	Subsample A	4.88	6803.500	5.85	< 0.001	0.41
	Subsample B	5.71				

Note. Mann-Whitney U tests were performed due to violations of normal distribution

### Factor structure of the BCAQ in an adolescent sample

Item-level descriptives and missing data information are provided in Supplemental Table. The overall rate of missing data was low (0.8%), with individual item missingness ranging from 0.7% (e.g. item 6) to 6.1% (item 9). The missingness pattern was primarily item-specific, with the majority of missing data (~ 52%) concentrated in two items (item 9 and item 21), likely due to their sensitive content. Regarding item-level acceptability, item means ranged from 1.60 to 3.14, and standard deviations ranged from 0.91 to 1.24, indicating no substantial floor or ceiling effects and sufficient variability across all items. Bartlett’s test of sphericity showed a significant result ($\:{\upchi\:}$^2^_(351)_ = 2544.95, *p* < .001). The overall Kaiser-Meyer-Olkin measure (0.90) also indicated that the data were suitable for factor analysis. Due to the sensitivity of Bartlett’s test of sphericity to non-normally distributed data, the anti-image matrices for each item were additionally checked for values < 0.5, with only values ≥ 0.67 (item 18) being found overall. The sampling adequacy indices therefore supported the suitability of the data for exploratory factor analysis. For an overview of descriptive statistics per item/subscale, see Supplemental Table 1.

The $\:{\upchi\:}$^2^ -test of CFA revealed significant deviations ($\:{\upchi\:}$^2^_(321)_ = 674.144, *p* < .001) from the three-factor structure tested in the model estimation for the given data. Besides an acceptable scaled value of $\:{\upchi\:}$^2^/df = 2.1 [57], the other fit indices also indicated a poor model fit (SRMR = 0.08; RMSEA = 0.09; RCFI = 0.83; RTLI = 0.81). Based on these findings, the original three-factor structure could not be replicated in the adolescent AN sample. Examination of the standardized residual matrix (see Supplemental Table 2) revealed several high positive deviations (>|1.96|), indicating that the model underestimated the relationships between the corresponding variables. For item-wise Spearman’s correlation analyses, see Supplemental Table 3.

For the subsequent EFA, principal axis factoring (PAF) and an oblique rotation method (promax) were selected as extraction methods due to violations of the normal distribution assumption (*n* = 138 observations, based on pairwise exclusion). Parallel analysis (based on factor eigenvalues) and visual evaluation of scree plots were performed to determine the number of factors. The EFA yielded a 3-factor structure with three factors explaining 52% of the variance (see Table 3). The first factor (“checking behavior”) explained the highest proportion of variance with 25%. However, the fit indices of the EFA also indicated only an acceptable model fit overall (RMSEA = 0.09, SRMR = 0.05, TLI = 0.82, CFI = 0.86, BIC = -787.69). Two items (item 18 and item 23) showed only low loadings (< 0.4) on any of the three factors and high uniqueness values (uniqueness item 18 = 0.93; uniqueness item 23 = 0.66; see Supplemental Table 4a and 4b). Regarding item discrimination, item 18 exhibited the lowest item-rest correlation (*r*_i, t_ of item 18 = 0.26; see Supplemental Table 5), indicating only marginal item discrimination [53]. In addition, items 9 and 21 (both items originally assigned to factor 2, “avoidance behavior”) deviated from the original structure of the BCAQ in the validation for adults with EDs and showed the highest loadings on factor 3 (“reassurance seeking”). Similarly, these items were among those with the lowest item-rest correlations (*r*_i, t_ of item 9 = 0.40 and *r*_i, t_ of item 21 = 0.45), had the highest proportion of missing values and were weaker indicators of the construct than the remaining items. Since all four items (item 9, item 18, item 21 and item 23) refer to physical/intimate contact, their elimination was deemed appropriate from both statistical and conceptual perspectives, as the nature of these questions may limit their suitability for an adolescent clinical sample. After eliminating these four items, the total proportion of explained variance increased to 56%. Except for the BIC (-565.48), the fit indices (RMSEA = 0.08, SRMR = 0.04, TLI = 0.87, CFI = 0.91) also improved, indicating a better fit of the model without the four eliminated items, although the overall model fit remained mixed. In this reduced model (23 items), the first factor (“checking behavior”; 12 items) and the third factor (“reassurance seeking”; 3 items) broadly corresponded to the factor structure reported in the adult validation study [33], whereas the eliminated items all belonged to factor 2 (“avoidance behavior”; reduced from 12 to 8 items). For scree plot analysis, see Supplemental Figure S1.

**Table 3:** Factor characteristics (“checking behavior”, “avoidance behavior” and “reassurance seeking”) before and after promax rotation

Factor		Before rotation			After rotation		
	Initial eigenvalues	SumSq. Loadings	% of variance explained	Cumulative %	SumSq. Loadings	% of variance explained	Cumulative %
1	11.02	10.58	39.18	39.18	6.70	24.83	24.83
2	2.52	2.07	7.66	46.84	4.78	17.71	42.54
3	1.84	1.35	5	51.84	2.51	9.30	51.84

With regard to reliability, the first factor (“checking behavior”) showed a Cronbach’s $\:\alpha\:$ = 0.94 and an item discrimination between *r*_i, t_ = 0.63 and *r*_i, t_ = 0.82 (*n* = 145). The second factor (“avoidance behavior”) showed a Cronbach’s $\:\alpha\:$ = 0.88 (with all items, *n* = 137; Cronbach’s $\:\alpha\:$ = 0.89 without the eliminated items, *n* = 146) and item discrimination between *r*_i, t_ = 0.26 and *r*_i, t_ = 0.72 (*r*_i, t_ = 0.53 and *r*_i, t_ = 0.75 without the eliminated items). The third factor (“reassurance seeking”) showed a Cronbach’s $\:\alpha\:$ = 0.78 and item discrimination between *r*_i, t_ = 0.54 and *r*_i, t_ = 0.67 (*n* = 141). Spearman correlations between the subscales “checking behavior” and “avoidance behavior” (*r* = .64, *p* < .001, *n* = 147; without the eliminated items: *r* = .63, *p* < .001, *n* = 147), “checking behavior” and “reassurance seeking” (*r* = .55, *p* < .001), and “avoidance behavior” and “reassurance seeking” (*r* = .36, *p* < .001, *n* = 144; without the eliminated items: *r* = .29, *p* < .001, *n* = 144) showed weak to strong significant correlations.

### Validity of the BCAQ in an adolescent sample

The Spearman correlation coefficients indicated moderate to strong correlations overall for the BCAQ, CDRS and EDE-Q subscales. For an overview of all pairwise Spearman correlation coefficients, see Table 4.

**Table 4:** Spearman correlation coefficients for BCAQ, CDRS and EDE-Q subscales

	Original, 27-items (*n* = 147)				Adapted, 23-items (*n* = 147)	
	BCAQ-CB	BCAQ-AB	BCAQ-RS	BCAQ-TS	BCAQ- AB	BCAQ- TS
**CDRS** (*n* = 91)						
Difference score felt – ideal	0.75	0.58	0.55	-	0.54	-
Difference score actual – ideal	0.65	0.51	0.44	-	0.49	-
**EDE-Q** (*n* = 144)						
Restraint	0.59	0.53	0.47	-	0.54	-
Eating concern	0.73	0.58	0.52	-	0.59	-
Weight concern	0.80	0.62	0.57	-	0.63	-
Shape concern	0.80	0.62	0.51	-	0.64	-
Total score	-	-	-	0.81	-	0.82

Note. Spearman’s rho values are reported. BCAQ-CB = “checking behavior”; BCAQ-AB = “avoidance behavior”; BCAQ-RS = “reassurance seeking”; BCAQ-TS = Total score. All correlations were significant at *p* < .001

With regard to the examination of known-groups validity for the original 27-item version, Kolmogorov-Smirnov tests revealed significantly different distributions (*p* < .05) between the adolescent and adult subsample for the BCAQ total score and the “avoidance behavior” subscale. No significant differences in distribution were found for the K-S tests of the subscales “checking behavior” (*p* = .135) and “reassurance seeking” (*p* = .979). For the BCAQ total score, there was a significant difference in the medians between the adolescent (*n* = 134, *Mdn* = 2.54) and adult sample (*n* = 68, *Mdn* = 2.67), *U* = 5445.500, *Z* = 2.7, *r* = .16, *p* < .05. Similarly, for the “avoidance behavior” subscale, there was a significant median difference between the adolescent (*n* = 134, *Mdn* = 2.25) and the adult (*n* = 68, *Mdn* = 2.83) sample, *U* = 6016.500, *Z* = 3.72, *r* = .26, *p* < .001. No significant differences were found between the groups for the subscales “checking behavior” (Adolescents: *n* = 134, *Mdn* = 2.88; Adults: *n* = 68, *Mdn* = 2.96, *U* = 5017.500, *Z* = 1.17, *r* = .08, *p* = .239), and “reassurance seeking” (Adolescents: *n* = 131, *Mdn* = 1.67; Adults: *n* = 68, *Mdn* = 1.83, *U* = 4816.000, *Z* = 0.95, *r* = .07, *p* = .340). For the final adapted 23-item version, the K-S tests indicated no significant distributional differences between adolescents and adults for the adapted BCAQ total score (*p* = .099) and the adapted “avoidance behavior” subscale (*p* = .169). The Mann-Whitney U test showed no significant median difference for the adapted BCAQ total score (Adolescents: *n* = 134, *Mdn* = 2.59; Adults: *n* = 68, *Mdn* = 2.65, *U* = 5184.000, *Z* = 1.60, *r* = .11, *p* = .110). A significant difference persisted for the “avoidance behavior” subscale (Adolescents: *n* = 134, *Mdn* = 2.38; Adults: *n* = 68, *Mdn* = 2.75, *U* = 5486.000, *Z* = 2.37, *r* = .17, *p* < .05). The other subscales remained non-significant.

## Discussion

The aim of the present study was to validate the BCAQ in a sample of adolescents with AN or atypical AN. Our analysis revealed a factor structure differing from the original version, consisting of 23 items and the three factors “avoidance behavior”, “checking behavior”, and “reassurance seeking”. The identified three-factor structure may facilitate a more differentiated assessment of body checking and avoidance behaviors. Distinguishing between avoidance behavior, checking behavior, and reassurance seeking may help to capture potentially distinct behavioral patterns that could be relevant for research and clinical practice. This divergent factor structure may be attributed to the younger age of the adolescent sample. The items eliminated due to low factor loadings (item 18 and item 23) or due to inconsistency with the a priori three factor structure (item 9 and item 21) primarily referred to sexual or intimate interactions with another person. Given that the mean age of the sample was 15 years, an age at which pubertal changes are ongoing and sexuality is still developing, this result appears plausible. Studies have shown that sexual behavior, desire, and readiness typically begin to emerge around this age [58]. However, developmental differences may not be the only explanation for the observed item performance. The inpatient treatment setting may also have influenced the factor loadings, as opportunities to engage in intimate or romantic relationships were generally restricted during treatment, both within the inpatient setting and with peers outside the clinic. In addition, AN itself may be associated with reduced sexual interest [59], which could further limit the relevance of these items in the present clinical sample. This may have resulted in restricted variability in responses to items addressing intimacy and sexuality. In addition, cultural influences cannot be ruled out, as cultural norms may affect adolescents’ willingness to disclose information about sexual behavior and intimate relationships [60]. Furthermore, developmental heterogeneity within adolescence should be taken into account. Previous research suggests that sexual interests and intimate relationships become increasingly relevant during mid- to late adolescence. Consequently, responses to these items may differ substantially between younger and older adolescents. Although the removal of these items improved the psychometric performance of the questionnaire in the present sample, it may also reduce the coverage of body checking and avoidance behaviors related to intimacy and sexuality. Previous studies have shown that a more positive body image is associated with greater sexual satisfaction [61, 62]. Therefore, these aspects may remain relevant, particularly for older adolescents, and should be further examined in future research before their exclusion from the questionnaire is considered definitive. It is also possible that these items capture aspects of interpersonal intimacy or avoidance of physical closeness that are only partially related to body checking and avoidance behaviors. This alternative interpretation should be explored in future studies to determine whether these items reflect a distinct, yet related, behavioral construct in female adolescent inpatients with AN or atypical AN. Future studies should also investigate whether these items may benefit from developmental adaptation rather than complete removal. With an explained variance of 56% and an overall mixed model fit, the adapted BCAQ appears to be a promising instrument for assessing body checking and avoidance behaviors in female adolescent inpatients with anorexia nervosa or atypical anorexia nervosa. To the best of our knowledge, this is the first study to examine the BCAQ in an adolescent population.

The reduced version of the questionnaire, comprising 23 items, demonstrated acceptable to excellent internal consistency. In the adolescent sample, item discrimination improved considerably after the four aforementioned items were removed. Regarding convergent validity, the BCAQ showed moderate to strong correlations with the subscales and total scores of the CDRS and EDE-Q. Known-groups validity was examined by comparing BCAQ scores between the adult and adolescent samples. Significant differences were observed for the “avoidance behavior” subscale. However, the corresponding effect size (Cohen’s r) was small, indicating that the magnitude of this group difference was modest. These differences can be explained by the greater symptom severity observed in the adult group. Adults exhibited substantially higher scores on both the BCAQ subscales and the EDE-Q, which may reflect the chronic course of EDs in adulthood. Since EDs typically onset during adolescence [63, 64], the adults in the present study likely had a longer illness duration and thus greater symptom severity [65]. This hypothesis could also provide a rationale for the significant discrepancy observed in the subscale “avoidance behavior” among adolescents and adults. However, given the substantially higher eating-disorder symptom severity in the adult group and the absence of established measurement invariance, these group differences should be interpreted as exploratory. Taken together, the findings suggest that the adapted 23-item BCAQ shows promising psychometric properties for assessing body checking and body avoidance behaviors in female adolescent inpatients with AN or atypical AN.

### Strengths and limitations

A notable strength of the present study is that it represents the first validation of the BCAQ in an adolescent sample with EDs. Furthermore, a range of psychometric properties were evaluated, including internal consistency and various aspects of validity. However, it is important to note that there are certain limitations that should be taken into consideration. A relevant limitation of the present study is the preliminary nature of the identified factor structure. The CFA indicated that the original three-factor structure of the BCAQ, previously established in an adult sample, could not be replicated in the present adolescent sample. Consequently, the adapted 23-item version derived from the EFA should be considered an exploratory factor solution. Due to the limited sample size, it was not feasible to confirm this factor structure using an independent validation sample or an appropriate split-sample approach. Therefore, further studies are needed to replicate and confirm the 23-item structure in independent samples of adolescents with AN or atypical AN. The cross-sectional design of the study limits the ability to draw conclusions about the temporal stability of the BCAQ. In addition, the study design did not allow the evaluation of test–retest reliability, responsiveness to treatment, longitudinal validity, or sensitivity to changes in symptom severity. These psychometric properties should be examined in future longitudinal studies before the questionnaire can be recommended for treatment monitoring or the assessment of clinical change. Furthermore, the sample consisted exclusively of female inpatients with AN or atypical AN. Therefore, the findings may not generalize to males, outpatients, adolescents with other ED diagnoses, or non-clinical adolescent populations. In addition, the modest sample size and the exploratory nature of the adapted 23-item factor structure warrant cautious interpretation of the findings. Replication in larger and more diverse adolescent samples is needed before the factor structure can be considered established. In addition, measurement invariance between adolescents and adults was not examined. Therefore, comparisons between both groups should be interpreted with caution until measurement invariance has been established in future studies. Moreover, the adult group showed substantially higher levels of ED symptomatology than the adolescent group, which may have contributed to the observed group differences. Therefore, these comparisons should be considered exploratory. In addition, further psychometric evaluation, including the assessment of discriminant validity using external measures, is warranted.

### Future directions

Further research is required to replicate the adapted 23-item factor structure in independent clinical and non-clinical adolescent samples. In addition, longitudinal studies should evaluate the stability of the questionnaire over time, including its test–retest reliability, responsiveness to treatment, and sensitivity to changes in symptom severity, before its use for treatment monitoring can be recommended.

## Conclusions

Findings of the present study provide preliminary evidence of internal consistency and cross-sectional construct validity for an exploratory 23-item version of the BCAQ in female adolescent inpatients with AN or atypical AN. However, as this factor structure was derived through EFA, it should be considered preliminary until it has been replicated and confirmed in independent samples of adolescents with anorexia nervosa or atypical anorexia nervosa.

## Supplementary information

Below is the link to the electronic supplementary material.

Supplementary Material 1

Supplementary Material 2

Supplementary Material 3

Supplementary Material 4

Supplementary Material 5

## Data Availability

The datasets generated and analyzed during the current study are available from the corresponding author upon reasonable request.

## Abbreviations

AN: Anorexia Nervosa
BA: Body Avoidance
BC: Body Checking
BCAQ: Body Checking and Avoidance Questionnaire
BCQ: Body Checking Questionnaire
BIAQ: Body Image Avoidance Questionnaire
BICI: Body Image Concern Inventory
BID: Body Image Distortion
BSQ: Body Shape Questionnaire
CDRS: Contour Drawing Rating Scale
CFA: Confirmatory Factor Analysis
ED: Eating Disorder
EDEQ: Eating Disorder Examination Questionnaire
EFA: Exploratory Factor Analysis
FIML: Full Information Maximum Likelihood
PAF: Principal Axis Factoring
RCFI: Comparative Fit Index
RTLI: Tucker-Lewis Index
RMSEA: Root Mean Square Error of Approximation
SRMR: Standardized Root Mean Square Residual
