# Supplementary material for: Measuring body checking and avoidance in adolescents: validation of a behavioral questionnaire
Source: BMC Psychol. 2026 Sep 18;14:1359. doi: 10.1186/s40359-026-05614-y (PMC13589350; doi:10.1186/s40359-026-05614-y)
Supplement: Supplementary file 1 [file 40359_2026_5614_MOESM1_ESM.docx]

**Supplemental Table 1.**
*Itemwise Descriptive Statistics*

|  | | | | | | | | | | | | | | | | | | | |
| --- | --- | --- | --- | --- | --- | --- | --- | --- | --- | --- | --- | --- | --- | --- | --- | --- | --- | --- | --- |
|  | | Valid | | Missing | | Median | | *M* | | *SD* | | Shapiro-Wilk | | P-value of Shapiro-Wilk | | Min | | Max | |
| Item_1 |  | 147 |  | 0 |  | 2.00 |  | 2.41 |  | 1.08 |  | 0.86 |  | < .001 |  | 1.00 |  | 4.00 |  |
| Item_2 |  | 147 |  | 0 |  | 3.00 |  | 2.93 |  | 1.11 |  | 0.81 |  | < .001 |  | 1.00 |  | 4.00 |  |
| Item_3 |  | 147 |  | 0 |  | 2.00 |  | 2.10 |  | 0.91 |  | 0.86 |  | < .001 |  | 1.00 |  | 4.00 |  |
| Item_4 |  | 147 |  | 0 |  | 3.00 |  | 3.03 |  | 1.11 |  | 0.78 |  | < .001 |  | 1.00 |  | 4.00 |  |
| Item_5 |  | 147 |  | 0 |  | 2.00 |  | 2.35 |  | 1.14 |  | 0.84 |  | < .001 |  | 1.00 |  | 4.00 |  |
| Item_6 |  | 146 |  | 1 |  | 3.00 |  | 2.55 |  | 1.02 |  | 0.87 |  | < .001 |  | 1.00 |  | 4.00 |  |
| Item_7 |  | 144 |  | 3 |  | 1.00 |  | 1.78 |  | 0.95 |  | 0.77 |  | < .001 |  | 1.00 |  | 4.00 |  |
| Item_8 |  | 147 |  | 0 |  | 2.00 |  | 2.42 |  | 1.24 |  | 0.81 |  | < .001 |  | 1.00 |  | 4.00 |  |
| Item_9 |  | 138 |  | 9 |  | 1.00 |  | 1.70 |  | 0.94 |  | 0.74 |  | < .001 |  | 1.00 |  | 4.00 |  |
| Item_10 |  | 147 |  | 0 |  | 2.00 |  | 2.01 |  | 1.09 |  | 0.80 |  | < .001 |  | 1.00 |  | 4.00 |  |
| Item_11 |  | 147 |  | 0 |  | 2.00 |  | 2.14 |  | 1.00 |  | 0.85 |  | < .001 |  | 1.00 |  | 4.00 |  |
| Item_12 |  | 147 |  | 0 |  | 3.00 |  | 2.73 |  | 1.10 |  | 0.84 |  | < .001 |  | 1.00 |  | 4.00 |  |
| Item_13 |  | 147 |  | 0 |  | 4.00 |  | 3.08 |  | 1.11 |  | 0.76 |  | < .001 |  | 1.00 |  | 4.00 |  |
| Item_14 |  | 147 |  | 0 |  | 3.00 |  | 2.77 |  | 1.16 |  | 0.82 |  | < .001 |  | 1.00 |  | 4.00 |  |
| Item_15 |  | 147 |  | 0 |  | 2.00 |  | 2.51 |  | 1.12 |  | 0.85 |  | < .001 |  | 1.00 |  | 4.00 |  |
| Item_16 |  | 147 |  | 0 |  | 3.00 |  | 2.81 |  | 1.06 |  | 0.85 |  | < .001 |  | 1.00 |  | 4.00 |  |
| Item_17 |  | 147 |  | 0 |  | 2.00 |  | 2.06 |  | 0.96 |  | 0.85 |  | < .001 |  | 1.00 |  | 4.00 |  |
| Item_18 |  | 146 |  | 1 |  | 3.00 |  | 2.63 |  | 1.02 |  | 0.87 |  | < .001 |  | 1.00 |  | 4.00 |  |
| Item_19 |  | 147 |  | 0 |  | 4.00 |  | 3.14 |  | 1.09 |  | 0.75 |  | < .001 |  | 1.00 |  | 4.00 |  |
| Item_20 |  | 144 |  | 3 |  | 2.00 |  | 2.00 |  | 1.15 |  | 0.77 |  | < .001 |  | 1.00 |  | 4.00 |  |
| Item_21 |  | 139 |  | 8 |  | 1.00 |  | 1.60 |  | 0.97 |  | 0.66 |  | < .001 |  | 1.00 |  | 4.00 |  |
| Item_22 |  | 141 |  | 6 |  | 2.00 |  | 2.05 |  | 1.14 |  | 0.79 |  | < .001 |  | 1.00 |  | 4.00 |  |
| Item_23 |  | 147 |  | 0 |  | 2.00 |  | 2.28 |  | 0.96 |  | 0.87 |  | < .001 |  | 1.00 |  | 4.00 |  |
| Item_24 |  | 145 |  | 2 |  | 2.00 |  | 2.15 |  | 1.16 |  | 0.81 |  | < .001 |  | 1.00 |  | 4.00 |  |
| Item_25 |  | 147 |  | 0 |  | 3.00 |  | 2.52 |  | 1.21 |  | 0.82 |  | < .001 |  | 1.00 |  | 4.00 |  |
| Item_26 |  | 147 |  | 0 |  | 2.00 |  | 2.18 |  | 1.10 |  | 0.84 |  | < .001 |  | 1.00 |  | 4.00 |  |
| Item_27 |  | 147 |  | 0 |  | 4.00 |  | 3.03 |  | 1.20 |  | 0.73 |  | < .001 |  | 1.00 |  | 4.00 |  |
| Checking behavior |  | 147 |  | 0 |  | 2.83 |  | 2.69 |  | 0.88 |  | 0.94 |  | < .001 |  | 1.00 |  | 4.00 |  |
| Avoidance behavior |  | 147 |  | 0 |  | 2.25 |  | 2.24 |  | 0.66 |  | 0.97 |  | 5.25×10^-3^ |  | 1.00 |  | 3.58 |  |
| Reassurance seeking |  | 144 |  | 3 |  | 1.67 |  | 1.94 |  | 0.91 |  | 0.88 |  | < .001 |  | 1.00 |  | 4.00 |  |
| Total score |  | 147 |  | 0 |  | 2.52 |  | 2.41 |  | 0.68 |  | 0.97 |  | < .001 |  | 1.04 |  | 3.74 |  |
|  | | | | | | | | | | | | | | | | | | | |
