# Supplementary material for: Measuring body checking and avoidance in adolescents: validation of a behavioral questionnaire
Source: BMC Psychol. 2026 Sep 18;14:1359. doi: 10.1186/s40359-026-05614-y (PMC13589350; doi:10.1186/s40359-026-05614-y)
Supplement: Supplementary file 2 [file 40359_2026_5614_MOESM2_ESM.docx]

**Supplemental Table 4a**.

*Factor loadings for each item of the BCAQ after promax rotation (pattern matrix). Loadings are only depicted, if they are greater than .40.*

|  | Items | Factor 1 | Factor 2 | Factor 3 | Uniqueness | *h*^2^ |
| --- | --- | --- | --- | --- | --- | --- |
| 1 | I avoid places like swimming lakes and beaches. |  | 0.74 |  | 0.38 | 0.62 |
| 2 | I test whether I can reach around my wrists and ankles with one hand. | 0.54 |  |  | 0.53 | 0.47 |
| 3 | I only wear dark, muted clothes or scarves to detract attention from my figure. |  | 0.66 |  | 0.50 | 0.50 |
| 4 | I touch certain body parts such as my stomach or my hips. | 0.76 |  |  | 0.41 | 0.59 |
| 5 | I make certain body movements to check whether my fat wobbles. | 0.64 |  |  | 0.44 | 0.56 |
| 6 | I do not show myself naked in front of other people, e.g., my partner, my family, or even at the doctor’s or the hospital. |  | 0.60 |  | 0.57 | 0.43 |
| 7 | I ask my partner or a girlfriend how attractive he/she finds me. |  |  | 0.54 | 0.63 | 0.37 |
| 8 | I measure the size of my thighs with my hands or with a measuring tape. | 0.62 |  |  | 0.49 | 0.51 |
| 9 | In sexual contact with a partner, I only adopt certain positions. |  |  | 0.68 | 0.53 | 0.47 |
| 10 | I pinch my skin together and measure the size of the folds. | 0.40 |  |  | 0.53 | 0.47 |
| 11 | I don’t wear any clothes which show my feminine curves, e.g., jeans or tight tops. |  | 0.69 |  | 0.54 | 0.46 |
| 12 | I avoid communal showers, saunas, swimming pools or spas. |  | 0.77 |  | 0.33 | 0.67 |
| 13 | I pull my stomach in to see what it’s like when it’s completely flat. | 0.77 |  |  | 0.33 | 0.67 |
| 14 | When I’m walking, I check whether my legs are rubbing together. | 0.88 |  |  | 0.25 | 0.75 |
| 15 | I don’t wear short clothing when exercising. |  | 0.74 |  | 0.49 | 0.51 |
| 16 | I compare my appearance with women from magazines or from the TV. | 0.70 |  |  | 0.51 | 0.49 |
| 17 | I wear clothes that cover my whole body, even in the summer. |  | 0.81 |  | 0.38 | 0.62 |
| 18 | I like being massaged and enjoy the physical contact. |  |  |  | 0.93 | 0.07 |
| 19 | In the mirror, I check whether my bones are visible. | 0.84 |  |  | 0.31 | 0.70 |
| 20 | I ask my partner or friends whether I have gained weight or should go on another diet. |  |  | 0.60 | 0.52 | 0.48 |
| 21 | I only have sex in the dark. |  |  | 0.65 | 0.52 | 0.48 |
| 22 | I ask my partner whether I look fat in certain clothes. |  |  | 0.69 | 0.41 | 0.59 |
| 23 | I avoid close physical contact with other people. |  |  |  | 0.66 | 0.34 |
| 24 | I check whether I have cellulite on my thighs when I’m sitting down. | 0.51 |  |  | 0.57 | 0.43 |
| 25 | I reach around my upper arm to measure how wide it is. | 0.55 |  |  | 0.38 | 0.62 |
| 26 | When I’m shopping, I don’t leave the changing room in the new clothes to look at myself publicly in the mirror. |  | 0.47 |  | 0.57 | 0.43 |
| 27 | I check in the mirror whether my thighs touch when I’m standing upright. | 0.87 |  |  | 0.29 | 0.71 |

**Supplemental Table 4b**.

*Factor loadings for each item of the BCAQ after promax rotation (structure matrix). Loadings are only depicted, if they are greater than .40.*

|  | Items | Factor 1 | Factor 2 | Factor 3 |
| --- | --- | --- | --- | --- |
| 1 | I avoid places like swimming lakes and beaches. | 0.52 | 0.75 |  |
| 2 | I test whether I can reach around my wrists and ankles with one hand. | 0.64 | 0.54 |  |
| 3 | I only wear dark, muted clothes or scarves to detract attention from my figure. | 0.45 | 0.70 |  |
| 4 | I touch certain body parts such as my stomach or my hips. | 0.77 | 0.42 | 0.43 |
| 5 | I make certain body movements to check whether my fat wobbles. | 0.74 | 0.45 | 0.52 |
| 6 | I do not show myself naked in front of other people, e.g., my partner, my family, or even at the doctor’s or the hospital. | 0.41 | 0.65 |  |
| 7 | I ask my partner or a girlfriend how attractive he/she finds me. | 0.42 |  | 0.59 |
| 8 | I measure the size of my thighs with my hands or with a measuring tape. | 0.71 | 0.47 | 0.45 |
| 9 | In sexual contact with a partner, I only adopt certain positions. |  |  | 0.68 |
| 10 | I pinch my skin together and measure the size of the folds. | 0.63 | 0.52 | 0.53 |
| 11 | I don’t wear any clothes which show my feminine curves, e.g., jeans or tight tops. |  | 0.68 |  |
| 12 | I avoid communal showers, saunas, swimming pools or spas. | 0.53 | 0.80 |  |
| 13 | I pull my stomach in to see what it’s like when it’s completely flat. | 0.82 | 0.51 | 0.47 |
| 14 | When I’m walking, I check whether my legs are rubbing together. | 0.87 | 0.48 | 0.46 |
| 15 | I don’t wear short clothing when exercising. |  | 0.72 |  |
| 16 | I compare my appearance with women from magazines or from the TV. | 0.68 |  |  |
| 17 | I wear clothes that cover my whole body, even in the summer. | 0.42 | 0.79 |  |
| 18 | I like being massaged and enjoy the physical contact. |  |  |  |
| 19 | In the mirror, I check whether my bones are visible. | 0.83 | 0.50 |  |
| 20 | I ask my partner or friends whether I have gained weight or should go on another diet. | 0.49 |  | 0.65 |
| 21 | I only have sex in the dark. |  | 0.44 | 0.64 |
| 22 | I ask my partner whether I look fat in certain clothes. | 0.51 |  | 0.72 |
| 23 | I avoid close physical contact with other people. | 0.42 | 0.53 | 0.47 |
| 24 | I check whether I have cellulite on my thighs when I’m sitting down. | 0.63 | 0.41 | 0.49 |
| 25 | I reach around my upper arm to measure how wide it is. | 0.74 | 0.65 | 0.45 |
| 26 | When I’m shopping, I don’t leave the changing room in the new clothes to look at myself publicly in the mirror. | 0.43 | 0.61 | 0.50 |
| 27 | I check in the mirror whether my thighs touch when I’m standing upright. | 0.84 | 0.46 | 0.42 |

*Figure S1.* Scree plot displays BCAQ version with 27 items. Parallel analysis and scree plot visual analysis support a three-factor structure, as three factor real data eigenvalues show higher values than simulated data from parallel analysis (based on FA).
