# Supplementary material for: Measuring body checking and avoidance in adolescents: validation of a behavioral questionnaire
Source: BMC Psychol. 2026 Sep 18;14:1359. doi: 10.1186/s40359-026-05614-y (PMC13589350; doi:10.1186/s40359-026-05614-y)
Supplement: Supplementary file 3 [file 40359_2026_5614_MOESM3_ESM.docx]

**Supplemental Table 5:**
*Reliability analyses and item characteristics*

**Checking behavior**

| *Frequentist Scale Reliability Statistics* | | | | | | | |
| --- | --- | --- | --- | --- | --- | --- | --- |
| Estimate | | Cronbach's α | | Average interitem correlation | | *M* | |
| Point estimate |  | 0.94 |  | 0.56 |  | 2.70 |  |
| 95% CI lower bound |  | 0.92 |  | 0.49 |  | 2.56 |  |
| 95% CI upper bound |  | 0.95 |  | 0.61 |  | 2.84 |  |
|  | | | | | | | |
| *Note.*   Of the observations, 145 complete cases were used. | | | | | | | |

| *Frequentist Individual Item Reliability Statistics* | | | | | | | | | |
| --- | --- | --- | --- | --- | --- | --- | --- | --- | --- |
|  | | If item dropped | |  | | | | | |
| Item | | Cronbach's α | | Item-rest correlation | | *M* | | *SD* | |
| BCAQ_Item_2 |  | 0.94 |  | 0.63 |  | 2.94 |  | 1.10 |  |
| BCAQ_Item_4 |  | 0.93 |  | 0.73 |  | 3.03 |  | 1.11 |  |
| BCAQ_Item_5 |  | 0.93 |  | 0.71 |  | 2.37 |  | 1.14 |  |
| BCAQ_Item_8 |  | 0.93 |  | 0.70 |  | 2.44 |  | 1.24 |  |
| BCAQ_Item_10 |  | 0.93 |  | 0.64 |  | 2.02 |  | 1.09 |  |
| BCAQ_Item_13 |  | 0.93 |  | 0.79 |  | 3.09 |  | 1.11 |  |
| BCAQ_Item_14 |  | 0.93 |  | 0.82 |  | 2.79 |  | 1.16 |  |
| BCAQ_Item_16 |  | 0.93 |  | 0.66 |  | 2.81 |  | 1.07 |  |
| BCAQ_Item_19 |  | 0.93 |  | 0.79 |  | 3.14 |  | 1.09 |  |
| BCAQ_Item_24 |  | 0.94 |  | 0.63 |  | 2.15 |  | 1.16 |  |
| BCAQ_Item_25 |  | 0.93 |  | 0.75 |  | 2.54 |  | 1.21 |  |
| BCAQ_Item_27 |  | 0.93 |  | 0.79 |  | 3.06 |  | 1.19 |  |
|  | | | | | | | | | |

**Avoidance behavior**

| *Frequentist Scale Reliability Statistics* | | | | | | | |
| --- | --- | --- | --- | --- | --- | --- | --- |
| Estimate | | Cronbach's α | | Average interitem correlation | | *M* | |
| Point estimate |  | 0.88 |  | 0.37 |  | 2.26 |  |
| 95% CI lower bound |  | 0.84 |  | 0.31 |  | 2.15 |  |
| 95% CI upper bound |  | 0.90 |  | 0.43 |  | 2.37 |  |
|  | | | | | | | |
| *Note.*   Of the observations, 137 complete cases were used. | | | | | | | |

| *Frequentist Individual Item Reliability Statistics* | | | | | | | | | |
| --- | --- | --- | --- | --- | --- | --- | --- | --- | --- |
|  | | If item dropped | |  | | | | | |
| Item | | Cronbach's α | | Item-rest correlation | | *M* | | *SD* | |
| BCAQ_Item_1 |  | 0.86 |  | 0.66 |  | 2.47 |  | 1.07 |  |
| BCAQ_Item_3 |  | 0.86 |  | 0.65 |  | 2.09 |  | 0.90 |  |
| BCAQ_Item_6 |  | 0.86 |  | 0.61 |  | 2.58 |  | 1.01 |  |
| BCAQ_Item_9 |  | 0.88 |  | 0.40 |  | 1.71 |  | 0.94 |  |
| BCAQ_Item_11 |  | 0.86 |  | 0.59 |  | 2.15 |  | 1.01 |  |
| BCAQ_Item_12 |  | 0.86 |  | 0.72 |  | 2.80 |  | 1.08 |  |
| BCAQ_Item_15 |  | 0.86 |  | 0.63 |  | 2.53 |  | 1.13 |  |
| BCAQ_Item_17 |  | 0.86 |  | 0.72 |  | 2.07 |  | 0.96 |  |
| BCAQ_Item_18 |  | 0.88 |  | 0.26 |  | 2.66 |  | 1.02 |  |
| BCAQ_Item_21 |  | 0.87 |  | 0.45 |  | 1.61 |  | 0.97 |  |
| BCAQ_Item_23 |  | 0.87 |  | 0.55 |  | 2.29 |  | 0.97 |  |
| BCAQ_Item_26 |  | 0.86 |  | 0.59 |  | 2.20 |  | 1.11 |  |
|  | | | | | | | | | |

**Avoidance behavior (without Item 9, 18, 21, 23)**

| *Frequentist Scale Reliability Statistics* | | | | | | | |
| --- | --- | --- | --- | --- | --- | --- | --- |
| Estimate | | Cronbach's α | | Average interitem correlation | | *M* | |
| Point estimate |  | 0.89 |  | 0.51 |  | 2.34 |  |
| 95% CI lower bound |  | 0.86 |  | 0.43 |  | 2.21 |  |
| 95% CI upper bound |  | 0.92 |  | 0.58 |  | 2.47 |  |
|  | | | | | | | |
| *Note.*   Of the observations, 146 complete cases were used. | | | | | | | |

| *Frequentist Individual Item Reliability Statistics* | | | | | | | | | |
| --- | --- | --- | --- | --- | --- | --- | --- | --- | --- |
|  | | If item dropped | |  | | | | | |
| Item | | Cronbach's α | | Item-rest correlation | | *M* | | *SD* | |
| BCAQ_Item_1 |  | 0.87 |  | 0.71 |  | 2.42 |  | 1.07 |  |
| BCAQ_Item_3 |  | 0.88 |  | 0.68 |  | 2.10 |  | 0.91 |  |
| BCAQ_Item_6 |  | 0.88 |  | 0.59 |  | 2.55 |  | 1.02 |  |
| BCAQ_Item_11 |  | 0.88 |  | 0.66 |  | 2.14 |  | 1.00 |  |
| BCAQ_Item_12 |  | 0.87 |  | 0.75 |  | 2.75 |  | 1.09 |  |
| BCAQ_Item_15 |  | 0.88 |  | 0.67 |  | 2.52 |  | 1.12 |  |
| BCAQ_Item_17 |  | 0.87 |  | 0.76 |  | 2.06 |  | 0.96 |  |
| BCAQ_Item_26 |  | 0.89 |  | 0.53 |  | 2.18 |  | 1.10 |  |
|  | | | | | | | | | |

**Reassurance seeking**

| *Frequentist Scale Reliability Statistics* | | | | | | | |
| --- | --- | --- | --- | --- | --- | --- | --- |
| Estimate | | Cronbach's α | | Average interitem correlation | | *M* | |
| Point estimate |  | 0.78 |  | 0.54 |  | 1.95 |  |
| 95% CI lower bound |  | 0.71 |  | 0.45 |  | 1.80 |  |
| 95% CI upper bound |  | 0.84 |  | 0.65 |  | 2.10 |  |
|  | | | | | | | |
| *Note.*   Of the observations, 141 complete cases were used. | | | | | | | |

| *Frequentist Individual Item Reliability Statistics* | | | | | | | | | |
| --- | --- | --- | --- | --- | --- | --- | --- | --- | --- |
|  | | If item dropped | |  | | | | | |
| Item | | Cronbach's α | | Item-rest correlation | | *M* | | *SD* | |
| BCAQ_Item_7 |  | 0.79 |  | 0.54 |  | 1.79 |  | 0.95 |  |
| BCAQ_Item_20 |  | 0.65 |  | 0.67 |  | 2.01 |  | 1.16 |  |
| BCAQ_Item_22 |  | 0.65 |  | 0.67 |  | 2.05 |  | 1.14 |  |
|  | | | | | | | | | |
