# Supplementary material for: Measuring body checking and avoidance in adolescents: validation of a behavioral questionnaire
Source: BMC Psychol. 2026 Sep 18;14:1359. doi: 10.1186/s40359-026-05614-y (PMC13589350; doi:10.1186/s40359-026-05614-y)
Supplement: Supplementary file 4 [file 40359_2026_5614_MOESM4_ESM.docx]

**Supplemental Table 2.**

*Matrix of standardized residuals*

|  | **Item 2** | **Item 4** | **Item 5** | **Item 8** | **Item 10** | **Item 13** | **Item 14** | **Item 16** | **Item 19** | **Item 24** | **Item 25** | **Item 27** | **Item 1** | **Item 3** | **Item 6** | **Item 9** | **Item 11** | **Item 12** | **Item 15** | **Item 17** | **Item 18** | **Item 21** | **Item 23** | **Item 26** | **Item 7** | **Item 20** | **Item 22** |
| --- | --- | --- | --- | --- | --- | --- | --- | --- | --- | --- | --- | --- | --- | --- | --- | --- | --- | --- | --- | --- | --- | --- | --- | --- | --- | --- | --- |
| **Item 2** | 0 | -0,17 | -0,83 | 0,48 | -0,17 | -0,44 | -0,37 | -0,93 | 1,32 | 0,38 | 1,37 | -0,53 | 1,28 | 0,89 | 1,60 | 0,22 | 0,88 | 1,03 | 1,60 | -0,24 | 0,40 | 0,51 | 0,16 | 1,17 | -0,42 | -0,32 | -1,46 |
| **Item 4** | -0,17 | 0 | 1,48 | -0,63 | 0,28 | 0,40 | -0,20 | -0,29 | 0,61 | -0,79 | -1,25 | 0,14 | 0,48 | 0,32 | 0,06 | 1,85 | -0,30 | -0,21 | **-2,23** | -1,51 | -0,68 | -0,54 | 0,72 | 0,07 | 0,87 | -0,67 | 0,63 |
| **Item 5** | -0,83 | 1,48 | 0 | 0,15 | **2,07** | 0,31 | -0,33 | -0,16 | -0,47 | 0,22 | -0,53 | -0,99 | -0,11 | 0,70 | 0,64 | 1,34 | -0,08 | -0,23 | -0,99 | -0,64 | -0,55 | -0,09 | 0,33 | 0,90 | 1,71 | 1,12 | 1,29 |
| **Item 8** | 0,48 | -0,63 | 0,15 | 0 | 0,81 | -0,89 | -0,02 | -0,20 | -0,86 | 0,36 | 1,60 | 0,32 | 0,09 | -1,02 | 0,21 | 1,35 | -1,57 | 0,46 | 0,14 | -0,53 | 0,01 | 0,34 | 1,56 | 1,22 | 0,13 | -0,22 | 0,32 |
| **Item 10** | -0,17 | 0,28 | **2,07** | 0,81 | 0 | -0,09 | -1,36 | 0,76 | -0,12 | 0,54 | 0,26 | -1,36 | 1,17 | 1,04 | 0,46 | **2,29** | 0,75 | 0,66 | 0,96 | 0,70 | 0,31 | **2,03** | 0,72 | 1,40 | 0,42 | 0,58 | 0,57 |
| **Item 13** | -0,44 | 0,40 | 0,31 | -0,89 | -0,09 | 0 | 0,40 | -0,59 | 0,01 | -1,16 | -0,61 | 0,81 | 0,25 | 0,34 | 0,03 | **2,37** | -0,51 | -0,09 | -0,04 | -1,17 | -0,85 | 0,18 | 0,13 | -0,06 | 0,33 | -0,37 | -0,14 |
| **Item 14** | -0,37 | -0,20 | -0,33 | -0,02 | -1,36 | 0,40 | 0 | 0,05 | -0,11 | -0,18 | -0,44 | 1,22 | -0,24 | -0,23 | -0,37 | 1,40 | -0,96 | -0,42 | -1,00 | -1,15 | -0,51 | -0,70 | 0,47 | 0,21 | 0,33 | 0,11 | 0,02 |
| **Item 16** | -0,93 | -0,29 | -0,16 | -0,20 | 0,76 | -0,59 | 0,05 | 0 | 0,93 | 0,75 | -0,02 | 0,18 | -0,59 | -0,15 | -0,42 | 0,18 | -0,89 | -0,54 | -0,84 | -0,74 | -0,57 | -0,66 | 0,63 | -0,63 | 1,21 | -0,38 | -0,06 |
| **Item 19** | 1,32 | 0,61 | -0,47 | -0,86 | -0,12 | 0,01 | -0,11 | 0,93 | 0 | -0,53 | 0,22 | -0,29 | -0,14 | -0,72 | 0,49 | 0,69 | 0,08 | 0,57 | -0,34 | -1,41 | -0,44 | -0,45 | -0,12 | 0,45 | -0,10 | -1,03 | -0,44 |
| **Item 24** | 0,38 | -0,79 | 0,22 | 0,36 | 0,54 | -1,16 | -0,18 | 0,75 | -0,53 | -0,07 | **2,00** | -0,83 | 0,14 | 0,64 | -0,74 | 0,57 | -0,96 | -0,51 | -0,74 | -0,02 | 1,87 | 0,60 | 0,64 | **1,99** | 1,10 | 1,90 | 0,90 |
| **Item 25** | 1,37 | -1,25 | -0,53 | 1,60 | 0,26 | -0,61 | -0,44 | -0,02 | 0,22 | **2,00** | 0 | -0,67 | 0,94 | 0,88 | 1,37 | 0,42 | 0,88 | **2,00** | 1,58 | 1,20 | 1,27 | 1,69 | 1,27 | **2,11** | -0,49 | -0,68 | -0,76 |
| **Item 27** | -0,53 | 0,14 | -0,99 | 0,32 | -1,36 | 0,81 | 1,22 | 0,18 | -0,29 | -0,83 | -0,67 | 0 | 0,04 | -0,58 | -0,79 | 1,42 | -1,16 | 0,10 | -1,64 | -1,04 | -0,29 | -0,76 | 1,37 | 0,09 | -0,41 | -0,15 | -0,35 |
| **Item 1** | 1,28 | 0,48 | -0,11 | 0,09 | 1,17 | 0,25 | -0,24 | -0,59 | -0,14 | 0,14 | 0,94 | 0,04 | 0 | 0,35 | -1,14 | -0,67 | -0,65 | 1,74 | 0,29 | 0,11 | -0,61 | -1,47 | -1,17 | -1,19 | 0,17 | -1,42 | -1,62 |
| **Item 3** | 0,89 | 0,32 | 0,70 | -1,02 | 1,04 | 0,34 | -0,23 | -0,15 | -0,72 | 0,64 | 0,88 | -0,58 | 0,35 | 0 | -0,92 | -0,07 | **2,29** | -1,51 | 0,98 | 0,93 | -0,40 | -0,90 | -0,96 | -0,56 | 1,11 | 0,10 | -0,37 |
| **Item 6** | 1,60 | 0,06 | 0,64 | 0,21 | 0,46 | 0,03 | -0,37 | -0,42 | 0,49 | -0,74 | 1,37 | -0,79 | -1,14 | -0,92 | 0,07 | -0,70 | -0,27 | 1,30 | 0,19 | -0,68 | 0,56 | 1,08 | 1,91 | 0,43 | 0,70 | 0,58 | -0,53 |
| **Item 9** | 0,22 | 1,85 | 1,34 | 1,35 | **2,29** | **2,37** | 1,40 | 0,18 | 0,69 | 0,57 | 0,42 | 1,42 | -0,67 | -0,07 | -0,70 | -0,15 | -1,14 | -0,65 | -0,21 | -1,29 | 0,41 | **4,57** | 1,43 | 0,63 | **3,87** | **2,03** | **3,87** |
| **Item 11** | 0,88 | -0,30 | -0,08 | -1,57 | 0,75 | -0,51 | -0,96 | -0,89 | 0,08 | -0,96 | 0,88 | -1,16 | -0,65 | **2,29** | -0,27 | -1,14 | 0 | -0,87 | 0,19 | 1,30 | -0,13 | -1,30 | -0,75 | 0,19 | 0,05 | -0,19 | -0,05 |
| **Item 12** | 1,03 | -0,21 | -0,23 | 0,46 | 0,66 | -0,09 | -0,42 | -0,54 | 0,57 | -0,51 | **2,00** | 0,10 | 1,74 | -1,51 | 1,30 | -0,65 | -0,87 | 0 | -0,37 | -0,56 | -0,96 | -0,27 | 0,80 | -0,39 | -0,35 | -0,83 | -0,96 |
| **Item 15** | 1,60 | **-2,23** | -0,99 | 0,14 | 0,96 | -0,04 | -1,00 | -0,84 | -0,34 | -0,74 | 1,58 | -1,64 | 0,29 | 0,98 | 0,19 | -0,21 | 0,19 | -0,37 | 0 | 0,59 | 0,09 | -0,03 | -1,46 | -0,51 | 1,27 | -0,65 | -0,56 |
| **Item 17** | -0,24 | -1,51 | -0,64 | -0,53 | 0,70 | -1,17 | -1,15 | -0,74 | -1,41 | -0,02 | 1,20 | -1,04 | 0,11 | 0,93 | -0,68 | -1,29 | 1,30 | -0,56 | 0,59 | 0 | 0,26 | -0,49 | -0,51 | 0,22 | 0,76 | -0,04 | -0,46 |
| **Item 18** | 0,40 | -0,68 | -0,55 | 0,01 | 0,31 | -0,85 | -0,51 | -0,57 | -0,44 | 1,87 | 1,27 | -0,29 | -0,61 | -0,40 | 0,56 | 0,41 | -0,13 | -0,96 | 0,09 | 0,26 | 0,02 | 1,13 | 1,57 | 1,64 | -0,70 | -0,24 | -0,26 |
| **Item 21** | 0,51 | -0,54 | -0,09 | 0,34 | **2,03** | 0,18 | -0,70 | -0,66 | -0,45 | 0,60 | 1,69 | -0,76 | -1,47 | -0,90 | 1,08 | **4,57** | -1,30 | -0,27 | -0,03 | -0,49 | 1,13 | -0,07 | 0,90 | 1,58 | 1,91 | **2,00** | 1,95 |
| **Item 23** | 0,16 | 0,72 | 0,33 | 1,56 | 0,72 | 0,13 | 0,47 | 0,63 | -0,12 | 0,64 | 1,27 | 1,37 | -1,17 | -0,96 | 1,91 | 1,43 | -0,75 | 0,80 | -1,46 | -0,51 | 1,57 | 0,90 | 0 | 1,73 | 1,31 | **2,11** | **1,97** |
| **Item 26** | 1,17 | 0,07 | 0,90 | 1,22 | 1,40 | -0,06 | 0,21 | -0,63 | 0,45 | **1,99** | **2,11** | 0,09 | -1,19 | -0,56 | 0,43 | 0,63 | 0,19 | -0,39 | -0,51 | 0,22 | 1,64 | 1,58 | 1,73 | 0 | 1,34 | 1,55 | 1,65 |
| **Item 7** | -0,42 | 0,87 | 1,71 | 0,13 | 0,42 | 0,33 | 0,33 | 1,21 | -0,10 | 1,10 | -0,49 | -0,41 | 0,17 | 1,11 | 0,70 | **3,87** | 0,05 | -0,35 | 1,27 | 0,76 | -0,70 | 1,91 | 1,31 | 1,34 | 0,00 | 0,08 | -0,16 |
| **Item 20** | -0,32 | -0,67 | 1,12 | -0,22 | 0,58 | -0,37 | 0,11 | -0,38 | -1,03 | 1,90 | -0,68 | -0,15 | -1,42 | 0,10 | 0,58 | **2,03** | -0,19 | -0,83 | -0,65 | -0,04 | -0,24 | **2,00** | **2,11** | 1,55 | 0,08 | 0,05 | 0,04 |
| **Item 22** | -1,46 | 0,63 | 1,29 | 0,32 | 0,57 | -0,14 | 0,02 | -0,06 | -0,44 | 0,90 | -0,76 | -0,35 | -1,62 | -0,37 | -0,53 | **3,87** | -0,05 | -0,96 | -0,56 | -0,46 | -0,26 | 1,95 | **1,97** | 1,65 | -0,16 | 0,04 | -0,08 |

*Note*. Values > |1.96| are highlighted, indicating deviations from the model.
