# Supplementary material for: Measuring body checking and avoidance in adolescents: validation of a behavioral questionnaire
Source: BMC Psychol. 2026 Sep 18;14:1359. doi: 10.1186/s40359-026-05614-y (PMC13589350; doi:10.1186/s40359-026-05614-y)
Supplement: Supplementary file 5 [file 40359_2026_5614_MOESM5_ESM.docx]

**Supplemental Table 3.**

| *Itemwise Spearman's Correlations* | | | | | | | | | | | |
| --- | --- | --- | --- | --- | --- | --- | --- | --- | --- | --- | --- |
|  | |  | |  | | n | | Spearman's rho | | p | |
| BCAQ_Item_1 |  | - |  | BCAQ_Item_2 |  | 147 |  | 0.45 | *** | < .001 |  |
| BCAQ_Item_1 |  | - |  | BCAQ_Item_3 |  | 147 |  | 0.60 | *** | < .001 |  |
| BCAQ_Item_1 |  | - |  | BCAQ_Item_4 |  | 147 |  | 0.43 | *** | < .001 |  |
| BCAQ_Item_1 |  | - |  | BCAQ_Item_5 |  | 147 |  | 0.38 | *** | < .001 |  |
| BCAQ_Item_1 |  | - |  | BCAQ_Item_6 |  | 146 |  | 0.39 | *** | < .001 |  |
| BCAQ_Item_1 |  | - |  | BCAQ_Item_7 |  | 144 |  | 0.19 | * | 0.03 |  |
| BCAQ_Item_1 |  | - |  | BCAQ_Item_8 |  | 147 |  | 0.38 | *** | < .001 |  |
| BCAQ_Item_1 |  | - |  | BCAQ_Item_9 |  | 138 |  | 0.21 | * | 0.01 |  |
| BCAQ_Item_1 |  | - |  | BCAQ_Item_10 |  | 147 |  | 0.45 | *** | < .001 |  |
| BCAQ_Item_1 |  | - |  | BCAQ_Item_11 |  | 147 |  | 0.48 | *** | < .001 |  |
| BCAQ_Item_1 |  | - |  | BCAQ_Item_12 |  | 147 |  | 0.76 | *** | < .001 |  |
| BCAQ_Item_1 |  | - |  | BCAQ_Item_13 |  | 147 |  | 0.45 | *** | < .001 |  |
| BCAQ_Item_1 |  | - |  | BCAQ_Item_14 |  | 147 |  | 0.42 | *** | < .001 |  |
| BCAQ_Item_1 |  | - |  | BCAQ_Item_15 |  | 147 |  | 0.57 | *** | < .001 |  |
| BCAQ_Item_1 |  | - |  | BCAQ_Item_16 |  | 147 |  | 0.29 | *** | < .001 |  |
| BCAQ_Item_1 |  | - |  | BCAQ_Item_17 |  | 147 |  | 0.62 | *** | < .001 |  |
| BCAQ_Item_1 |  | - |  | BCAQ_Item_18 |  | 146 |  | **0.11** |  | 0.19 |  |
| BCAQ_Item_1 |  | - |  | BCAQ_Item_19 |  | 147 |  | 0.41 | *** | < .001 |  |
| BCAQ_Item_1 |  | - |  | BCAQ_Item_20 |  | 144 |  | **0.09** |  | 0.27 |  |
| BCAQ_Item_1 |  | - |  | BCAQ_Item_21 |  | 139 |  | 0.20 | * | 0.02 |  |
| BCAQ_Item_1 |  | - |  | BCAQ_Item_22 |  | 141 |  | **0.07** |  | 0.38 |  |
| BCAQ_Item_1 |  | - |  | BCAQ_Item_23 |  | 147 |  | 0.35 | *** | < .001 |  |
| BCAQ_Item_1 |  | - |  | BCAQ_Item_24 |  | 145 |  | 0.36 | *** | < .001 |  |
| BCAQ_Item_1 |  | - |  | BCAQ_Item_25 |  | 147 |  | 0.47 | *** | < .001 |  |
| BCAQ_Item_1 |  | - |  | BCAQ_Item_26 |  | 147 |  | 0.37 | *** | < .001 |  |
| BCAQ_Item_1 |  | - |  | BCAQ_Item_27 |  | 147 |  | 0.46 | *** | < .001 |  |
| BCAQ_Item_2 |  | - |  | BCAQ_Item_3 |  | 147 |  | 0.40 | *** | < .001 |  |
| BCAQ_Item_2 |  | - |  | BCAQ_Item_4 |  | 147 |  | 0.48 | *** | < .001 |  |
| BCAQ_Item_2 |  | - |  | BCAQ_Item_5 |  | 147 |  | 0.41 | *** | < .001 |  |
| BCAQ_Item_2 |  | - |  | BCAQ_Item_6 |  | 146 |  | 0.39 | *** | < .001 |  |
| BCAQ_Item_2 |  | - |  | BCAQ_Item_7 |  | 144 |  | 0.21 | * | 0.01 |  |
| BCAQ_Item_2 |  | - |  | BCAQ_Item_8 |  | 147 |  | 0.52 | *** | < .001 |  |
| BCAQ_Item_2 |  | - |  | BCAQ_Item_9 |  | 138 |  | **0.16** |  | 0.06 |  |
| BCAQ_Item_2 |  | - |  | BCAQ_Item_10 |  | 147 |  | 0.45 | *** | < .001 |  |
| BCAQ_Item_2 |  | - |  | BCAQ_Item_11 |  | 147 |  | 0.40 | *** | < .001 |  |
| BCAQ_Item_2 |  | - |  | BCAQ_Item_12 |  | 147 |  | 0.46 | *** | < .001 |  |
| BCAQ_Item_2 |  | - |  | BCAQ_Item_13 |  | 147 |  | 0.50 | *** | < .001 |  |
| BCAQ_Item_2 |  | - |  | BCAQ_Item_14 |  | 147 |  | 0.52 | *** | < .001 |  |
| BCAQ_Item_2 |  | - |  | BCAQ_Item_15 |  | 147 |  | 0.44 | *** | < .001 |  |
| BCAQ_Item_2 |  | - |  | BCAQ_Item_16 |  | 147 |  | 0.36 | *** | < .001 |  |
| BCAQ_Item_2 |  | - |  | BCAQ_Item_17 |  | 147 |  | 0.34 | *** | < .001 |  |
| BCAQ_Item_2 |  | - |  | BCAQ_Item_18 |  | 146 |  | **0.13** |  | 0.11 |  |
| BCAQ_Item_2 |  | - |  | BCAQ_Item_19 |  | 147 |  | 0.67 | *** | < .001 |  |
| BCAQ_Item_2 |  | - |  | BCAQ_Item_20 |  | 144 |  | 0.31 | *** | < .001 |  |
| BCAQ_Item_2 |  | - |  | BCAQ_Item_21 |  | 139 |  | 0.21 | * | 0.01 |  |
| BCAQ_Item_2 |  | - |  | BCAQ_Item_22 |  | 141 |  | 0.22 | ** | 9.26×10^-3^ |  |
| BCAQ_Item_2 |  | - |  | BCAQ_Item_23 |  | 147 |  | 0.26 | ** | 1.65×10^-3^ |  |
| BCAQ_Item_2 |  | - |  | BCAQ_Item_24 |  | 145 |  | 0.48 | *** | < .001 |  |
| BCAQ_Item_2 |  | - |  | BCAQ_Item_25 |  | 147 |  | 0.62 | *** | < .001 |  |
| BCAQ_Item_2 |  | - |  | BCAQ_Item_26 |  | 147 |  | 0.37 | *** | < .001 |  |
| BCAQ_Item_2 |  | - |  | BCAQ_Item_27 |  | 147 |  | 0.55 | *** | < .001 |  |
| BCAQ_Item_3 |  | - |  | BCAQ_Item_4 |  | 147 |  | 0.40 | *** | < .001 |  |
| BCAQ_Item_3 |  | - |  | BCAQ_Item_5 |  | 147 |  | 0.42 | *** | < .001 |  |
| BCAQ_Item_3 |  | - |  | BCAQ_Item_6 |  | 146 |  | 0.39 | *** | < .001 |  |
| BCAQ_Item_3 |  | - |  | BCAQ_Item_7 |  | 144 |  | 0.25 | ** | 2.29×10^-3^ |  |
| BCAQ_Item_3 |  | - |  | BCAQ_Item_8 |  | 147 |  | 0.28 | *** | < .001 |  |
| BCAQ_Item_3 |  | - |  | BCAQ_Item_9 |  | 138 |  | 0.31 | *** | < .001 |  |
| BCAQ_Item_3 |  | - |  | BCAQ_Item_10 |  | 147 |  | 0.44 | *** | < .001 |  |
| BCAQ_Item_3 |  | - |  | BCAQ_Item_11 |  | 147 |  | 0.68 | *** | < .001 |  |
| BCAQ_Item_3 |  | - |  | BCAQ_Item_12 |  | 147 |  | 0.46 | *** | < .001 |  |
| BCAQ_Item_3 |  | - |  | BCAQ_Item_13 |  | 147 |  | 0.44 | *** | < .001 |  |
| BCAQ_Item_3 |  | - |  | BCAQ_Item_14 |  | 147 |  | 0.41 | *** | < .001 |  |
| BCAQ_Item_3 |  | - |  | BCAQ_Item_15 |  | 147 |  | 0.58 | *** | < .001 |  |
| BCAQ_Item_3 |  | - |  | BCAQ_Item_16 |  | 147 |  | 0.32 | *** | < .001 |  |
| BCAQ_Item_3 |  | - |  | BCAQ_Item_17 |  | 147 |  | 0.65 | *** | < .001 |  |
| BCAQ_Item_3 |  | - |  | BCAQ_Item_18 |  | 146 |  | **0.11** |  | 0.18 |  |
| BCAQ_Item_3 |  | - |  | BCAQ_Item_19 |  | 147 |  | 0.36 | *** | < .001 |  |
| BCAQ_Item_3 |  | - |  | BCAQ_Item_20 |  | 144 |  | 0.21 | * | 0.01 |  |
| BCAQ_Item_3 |  | - |  | BCAQ_Item_21 |  | 139 |  | 0.28 | *** | < .001 |  |
| BCAQ_Item_3 |  | - |  | BCAQ_Item_22 |  | 141 |  | 0.18 | * | 0.04 |  |
| BCAQ_Item_3 |  | - |  | BCAQ_Item_23 |  | 147 |  | 0.33 | *** | < .001 |  |
| BCAQ_Item_3 |  | - |  | BCAQ_Item_24 |  | 145 |  | 0.37 | *** | < .001 |  |
| BCAQ_Item_3 |  | - |  | BCAQ_Item_25 |  | 147 |  | 0.44 | *** | < .001 |  |
| BCAQ_Item_3 |  | - |  | BCAQ_Item_26 |  | 147 |  | 0.40 | *** | < .001 |  |
| BCAQ_Item_3 |  | - |  | BCAQ_Item_27 |  | 147 |  | 0.39 | *** | < .001 |  |
| BCAQ_Item_4 |  | - |  | BCAQ_Item_5 |  | 147 |  | 0.67 | *** | < .001 |  |
| BCAQ_Item_4 |  | - |  | BCAQ_Item_6 |  | 146 |  | 0.33 | *** | < .001 |  |
| BCAQ_Item_4 |  | - |  | BCAQ_Item_7 |  | 144 |  | 0.39 | *** | < .001 |  |
| BCAQ_Item_4 |  | - |  | BCAQ_Item_8 |  | 147 |  | 0.50 | *** | < .001 |  |
| BCAQ_Item_4 |  | - |  | BCAQ_Item_9 |  | 138 |  | 0.38 | *** | < .001 |  |
| BCAQ_Item_4 |  | - |  | BCAQ_Item_10 |  | 147 |  | 0.55 | *** | < .001 |  |
| BCAQ_Item_4 |  | - |  | BCAQ_Item_11 |  | 147 |  | 0.34 | *** | < .001 |  |
| BCAQ_Item_4 |  | - |  | BCAQ_Item_12 |  | 147 |  | 0.38 | *** | < .001 |  |
| BCAQ_Item_4 |  | - |  | BCAQ_Item_13 |  | 147 |  | 0.66 | *** | < .001 |  |
| BCAQ_Item_4 |  | - |  | BCAQ_Item_14 |  | 147 |  | 0.63 | *** | < .001 |  |
| BCAQ_Item_4 |  | - |  | BCAQ_Item_15 |  | 147 |  | 0.19 | * | 0.02 |  |
| BCAQ_Item_4 |  | - |  | BCAQ_Item_16 |  | 147 |  | 0.46 | *** | < .001 |  |
| BCAQ_Item_4 |  | - |  | BCAQ_Item_17 |  | 147 |  | 0.30 | *** | < .001 |  |
| BCAQ_Item_4 |  | - |  | BCAQ_Item_18 |  | 146 |  | **0.06** |  | 0.45 |  |
| BCAQ_Item_4 |  | - |  | BCAQ_Item_19 |  | 147 |  | 0.65 | *** | < .001 |  |
| BCAQ_Item_4 |  | - |  | BCAQ_Item_20 |  | 144 |  | 0.35 | *** | < .001 |  |
| BCAQ_Item_4 |  | - |  | BCAQ_Item_21 |  | 139 |  | 0.20 | * | 0.02 |  |
| BCAQ_Item_4 |  | - |  | BCAQ_Item_22 |  | 141 |  | 0.44 | *** | < .001 |  |
| BCAQ_Item_4 |  | - |  | BCAQ_Item_23 |  | 147 |  | 0.36 | *** | < .001 |  |
| BCAQ_Item_4 |  | - |  | BCAQ_Item_24 |  | 145 |  | 0.43 | *** | < .001 |  |
| BCAQ_Item_4 |  | - |  | BCAQ_Item_25 |  | 147 |  | 0.47 | *** | < .001 |  |
| BCAQ_Item_4 |  | - |  | BCAQ_Item_26 |  | 147 |  | 0.34 | *** | < .001 |  |
| BCAQ_Item_4 |  | - |  | BCAQ_Item_27 |  | 147 |  | 0.65 | *** | < .001 |  |
| BCAQ_Item_5 |  | - |  | BCAQ_Item_6 |  | 146 |  | 0.37 | *** | < .001 |  |
| BCAQ_Item_5 |  | - |  | BCAQ_Item_7 |  | 144 |  | 0.43 | *** | < .001 |  |
| BCAQ_Item_5 |  | - |  | BCAQ_Item_8 |  | 147 |  | 0.54 | *** | < .001 |  |
| BCAQ_Item_5 |  | - |  | BCAQ_Item_9 |  | 138 |  | 0.32 | *** | < .001 |  |
| BCAQ_Item_5 |  | - |  | BCAQ_Item_10 |  | 147 |  | 0.64 | *** | < .001 |  |
| BCAQ_Item_5 |  | - |  | BCAQ_Item_11 |  | 147 |  | 0.34 | *** | < .001 |  |
| BCAQ_Item_5 |  | - |  | BCAQ_Item_12 |  | 147 |  | 0.38 | *** | < .001 |  |
| BCAQ_Item_5 |  | - |  | BCAQ_Item_13 |  | 147 |  | 0.63 | *** | < .001 |  |
| BCAQ_Item_5 |  | - |  | BCAQ_Item_14 |  | 147 |  | 0.60 | *** | < .001 |  |
| BCAQ_Item_5 |  | - |  | BCAQ_Item_15 |  | 147 |  | 0.27 | *** | < .001 |  |
| BCAQ_Item_5 |  | - |  | BCAQ_Item_16 |  | 147 |  | 0.48 | *** | < .001 |  |
| BCAQ_Item_5 |  | - |  | BCAQ_Item_17 |  | 147 |  | 0.37 | *** | < .001 |  |
| BCAQ_Item_5 |  | - |  | BCAQ_Item_18 |  | 146 |  | **0.06** |  | 0.50 |  |
| BCAQ_Item_5 |  | - |  | BCAQ_Item_19 |  | 147 |  | 0.58 | *** | < .001 |  |
| BCAQ_Item_5 |  | - |  | BCAQ_Item_20 |  | 144 |  | 0.47 | *** | < .001 |  |
| BCAQ_Item_5 |  | - |  | BCAQ_Item_21 |  | 139 |  | 0.22 | ** | 8.57×10^-3^ |  |
| BCAQ_Item_5 |  | - |  | BCAQ_Item_22 |  | 141 |  | 0.46 | *** | < .001 |  |
| BCAQ_Item_5 |  | - |  | BCAQ_Item_23 |  | 147 |  | 0.31 | *** | < .001 |  |
| BCAQ_Item_5 |  | - |  | BCAQ_Item_24 |  | 145 |  | 0.48 | *** | < .001 |  |
| BCAQ_Item_5 |  | - |  | BCAQ_Item_25 |  | 147 |  | 0.52 | *** | < .001 |  |
| BCAQ_Item_5 |  | - |  | BCAQ_Item_26 |  | 147 |  | 0.38 | *** | < .001 |  |
| BCAQ_Item_5 |  | - |  | BCAQ_Item_27 |  | 147 |  | 0.55 | *** | < .001 |  |
| BCAQ_Item_6 |  | - |  | BCAQ_Item_7 |  | 143 |  | 0.18 | * | 0.03 |  |
| BCAQ_Item_6 |  | - |  | BCAQ_Item_8 |  | 146 |  | 0.33 | *** | < .001 |  |
| BCAQ_Item_6 |  | - |  | BCAQ_Item_9 |  | 137 |  | 0.20 | * | 0.02 |  |
| BCAQ_Item_6 |  | - |  | BCAQ_Item_10 |  | 146 |  | 0.33 | *** | < .001 |  |
| BCAQ_Item_6 |  | - |  | BCAQ_Item_11 |  | 146 |  | 0.43 | *** | < .001 |  |
| BCAQ_Item_6 |  | - |  | BCAQ_Item_12 |  | 146 |  | 0.61 | *** | < .001 |  |
| BCAQ_Item_6 |  | - |  | BCAQ_Item_13 |  | 146 |  | 0.36 | *** | < .001 |  |
| BCAQ_Item_6 |  | - |  | BCAQ_Item_14 |  | 146 |  | 0.34 | *** | < .001 |  |
| BCAQ_Item_6 |  | - |  | BCAQ_Item_15 |  | 146 |  | 0.46 | *** | < .001 |  |
| BCAQ_Item_6 |  | - |  | BCAQ_Item_16 |  | 146 |  | 0.24 | ** | 3.02×10^-3^ |  |
| BCAQ_Item_6 |  | - |  | BCAQ_Item_17 |  | 146 |  | 0.47 | *** | < .001 |  |
| BCAQ_Item_6 |  | - |  | BCAQ_Item_18 |  | 145 |  | 0.18 | * | 0.03 |  |
| BCAQ_Item_6 |  | - |  | BCAQ_Item_19 |  | 146 |  | 0.40 | *** | < .001 |  |
| BCAQ_Item_6 |  | - |  | BCAQ_Item_20 |  | 143 |  | 0.22 | ** | 8.13×10^-3^ |  |
| BCAQ_Item_6 |  | - |  | BCAQ_Item_21 |  | 138 |  | 0.36 | *** | < .001 |  |
| BCAQ_Item_6 |  | - |  | BCAQ_Item_22 |  | 140 |  | **0.15** |  | 0.08 |  |
| BCAQ_Item_6 |  | - |  | BCAQ_Item_23 |  | 146 |  | 0.51 | *** | < .001 |  |
| BCAQ_Item_6 |  | - |  | BCAQ_Item_24 |  | 144 |  | 0.21 | * | 0.01 |  |
| BCAQ_Item_6 |  | - |  | BCAQ_Item_25 |  | 146 |  | 0.43 | *** | < .001 |  |
| BCAQ_Item_6 |  | - |  | BCAQ_Item_26 |  | 146 |  | 0.43 | *** | < .001 |  |
| BCAQ_Item_6 |  | - |  | BCAQ_Item_27 |  | 146 |  | 0.31 | *** | < .001 |  |
| BCAQ_Item_7 |  | - |  | BCAQ_Item_8 |  | 144 |  | 0.31 | *** | < .001 |  |
| BCAQ_Item_7 |  | - |  | BCAQ_Item_9 |  | 135 |  | 0.49 | *** | < .001 |  |
| BCAQ_Item_7 |  | - |  | BCAQ_Item_10 |  | 144 |  | 0.30 | *** | < .001 |  |
| BCAQ_Item_7 |  | - |  | BCAQ_Item_11 |  | 144 |  | **0.16** |  | 0.06 |  |
| BCAQ_Item_7 |  | - |  | BCAQ_Item_12 |  | 144 |  | **0.16** |  | 0.06 |  |
| BCAQ_Item_7 |  | - |  | BCAQ_Item_13 |  | 144 |  | 0.35 | *** | < .001 |  |
| BCAQ_Item_7 |  | - |  | BCAQ_Item_14 |  | 144 |  | 0.35 | *** | < .001 |  |
| BCAQ_Item_7 |  | - |  | BCAQ_Item_15 |  | 144 |  | 0.23 | ** | 5.05×10^-3^ |  |
| BCAQ_Item_7 |  | - |  | BCAQ_Item_16 |  | 144 |  | 0.34 | *** | < .001 |  |
| BCAQ_Item_7 |  | - |  | BCAQ_Item_17 |  | 144 |  | 0.24 | ** | 4.54×10^-3^ |  |
| BCAQ_Item_7 |  | - |  | BCAQ_Item_18 |  | 143 |  | **-0.00967** |  | 0.91 |  |
| BCAQ_Item_7 |  | - |  | BCAQ_Item_19 |  | 144 |  | 0.29 | *** | < .001 |  |
| BCAQ_Item_7 |  | - |  | BCAQ_Item_20 |  | 144 |  | 0.51 | *** | < .001 |  |
| BCAQ_Item_7 |  | - |  | BCAQ_Item_21 |  | 136 |  | 0.35 | *** | < .001 |  |
| BCAQ_Item_7 |  | - |  | BCAQ_Item_22 |  | 141 |  | 0.53 | *** | < .001 |  |
| BCAQ_Item_7 |  | - |  | BCAQ_Item_23 |  | 144 |  | 0.24 | ** | 4.57×10^-3^ |  |
| BCAQ_Item_7 |  | - |  | BCAQ_Item_24 |  | 142 |  | 0.34 | *** | < .001 |  |
| BCAQ_Item_7 |  | - |  | BCAQ_Item_25 |  | 144 |  | 0.27 | *** | < .001 |  |
| BCAQ_Item_7 |  | - |  | BCAQ_Item_26 |  | 144 |  | 0.26 | ** | 1.74×10^-3^ |  |
| BCAQ_Item_7 |  | - |  | BCAQ_Item_27 |  | 144 |  | 0.31 | *** | < .001 |  |
| BCAQ_Item_8 |  | - |  | BCAQ_Item_9 |  | 138 |  | 0.31 | *** | < .001 |  |
| BCAQ_Item_8 |  | - |  | BCAQ_Item_10 |  | 147 |  | 0.54 | *** | < .001 |  |
| BCAQ_Item_8 |  | - |  | BCAQ_Item_11 |  | 147 |  | 0.23 | ** | 4.96×10^-3^ |  |
| BCAQ_Item_8 |  | - |  | BCAQ_Item_12 |  | 147 |  | 0.43 | *** | < .001 |  |
| BCAQ_Item_8 |  | - |  | BCAQ_Item_13 |  | 147 |  | 0.54 | *** | < .001 |  |
| BCAQ_Item_8 |  | - |  | BCAQ_Item_14 |  | 147 |  | 0.61 | *** | < .001 |  |
| BCAQ_Item_8 |  | - |  | BCAQ_Item_15 |  | 147 |  | 0.36 | *** | < .001 |  |
| BCAQ_Item_8 |  | - |  | BCAQ_Item_16 |  | 147 |  | 0.46 | *** | < .001 |  |
| BCAQ_Item_8 |  | - |  | BCAQ_Item_17 |  | 147 |  | 0.37 | *** | < .001 |  |
| BCAQ_Item_8 |  | - |  | BCAQ_Item_18 |  | 146 |  | **0.10** |  | 0.22 |  |
| BCAQ_Item_8 |  | - |  | BCAQ_Item_19 |  | 147 |  | 0.56 | *** | < .001 |  |
| BCAQ_Item_8 |  | - |  | BCAQ_Item_20 |  | 144 |  | 0.34 | *** | < .001 |  |
| BCAQ_Item_8 |  | - |  | BCAQ_Item_21 |  | 139 |  | 0.24 | ** | 3.75×10^-3^ |  |
| BCAQ_Item_8 |  | - |  | BCAQ_Item_22 |  | 141 |  | 0.40 | *** | < .001 |  |
| BCAQ_Item_8 |  | - |  | BCAQ_Item_23 |  | 147 |  | 0.40 | *** | < .001 |  |
| BCAQ_Item_8 |  | - |  | BCAQ_Item_24 |  | 145 |  | 0.50 | *** | < .001 |  |
| BCAQ_Item_8 |  | - |  | BCAQ_Item_25 |  | 147 |  | 0.66 | *** | < .001 |  |
| BCAQ_Item_8 |  | - |  | BCAQ_Item_26 |  | 147 |  | 0.40 | *** | < .001 |  |
| BCAQ_Item_8 |  | - |  | BCAQ_Item_27 |  | 147 |  | 0.65 | *** | < .001 |  |
| BCAQ_Item_9 |  | - |  | BCAQ_Item_10 |  | 138 |  | 0.40 | *** | < .001 |  |
| BCAQ_Item_9 |  | - |  | BCAQ_Item_11 |  | 138 |  | 0.19 | * | 0.03 |  |
| BCAQ_Item_9 |  | - |  | BCAQ_Item_12 |  | 138 |  | 0.24 | ** | 4.01×10^-3^ |  |
| BCAQ_Item_9 |  | - |  | BCAQ_Item_13 |  | 138 |  | 0.44 | *** | < .001 |  |
| BCAQ_Item_9 |  | - |  | BCAQ_Item_14 |  | 138 |  | 0.34 | *** | < .001 |  |
| BCAQ_Item_9 |  | - |  | BCAQ_Item_15 |  | 138 |  | 0.26 | ** | 2.30×10^-3^ |  |
| BCAQ_Item_9 |  | - |  | BCAQ_Item_16 |  | 138 |  | 0.23 | ** | 6.45×10^-3^ |  |
| BCAQ_Item_9 |  | - |  | BCAQ_Item_17 |  | 138 |  | 0.23 | ** | 5.92×10^-3^ |  |
| BCAQ_Item_9 |  | - |  | BCAQ_Item_18 |  | 138 |  | **0.13** |  | 0.12 |  |
| BCAQ_Item_9 |  | - |  | BCAQ_Item_19 |  | 138 |  | 0.27 | ** | 1.36×10^-3^ |  |
| BCAQ_Item_9 |  | - |  | BCAQ_Item_20 |  | 135 |  | 0.32 | *** | < .001 |  |
| BCAQ_Item_9 |  | - |  | BCAQ_Item_21 |  | 138 |  | 0.66 | *** | < .001 |  |
| BCAQ_Item_9 |  | - |  | BCAQ_Item_22 |  | 135 |  | 0.47 | *** | < .001 |  |
| BCAQ_Item_9 |  | - |  | BCAQ_Item_23 |  | 138 |  | 0.33 | *** | < .001 |  |
| BCAQ_Item_9 |  | - |  | BCAQ_Item_24 |  | 138 |  | 0.24 | ** | 3.96×10^-3^ |  |
| BCAQ_Item_9 |  | - |  | BCAQ_Item_25 |  | 138 |  | 0.27 | ** | 1.55×10^-3^ |  |
| BCAQ_Item_9 |  | - |  | BCAQ_Item_26 |  | 138 |  | 0.32 | *** | < .001 |  |
| BCAQ_Item_9 |  | - |  | BCAQ_Item_27 |  | 138 |  | 0.34 | *** | < .001 |  |
| BCAQ_Item_10 |  | - |  | BCAQ_Item_11 |  | 147 |  | 0.39 | *** | < .001 |  |
| BCAQ_Item_10 |  | - |  | BCAQ_Item_12 |  | 147 |  | 0.42 | *** | < .001 |  |
| BCAQ_Item_10 |  | - |  | BCAQ_Item_13 |  | 147 |  | 0.57 | *** | < .001 |  |
| BCAQ_Item_10 |  | - |  | BCAQ_Item_14 |  | 147 |  | 0.47 | *** | < .001 |  |
| BCAQ_Item_10 |  | - |  | BCAQ_Item_15 |  | 147 |  | 0.41 | *** | < .001 |  |
| BCAQ_Item_10 |  | - |  | BCAQ_Item_16 |  | 147 |  | 0.49 | *** | < .001 |  |
| BCAQ_Item_10 |  | - |  | BCAQ_Item_17 |  | 147 |  | 0.46 | *** | < .001 |  |
| BCAQ_Item_10 |  | - |  | BCAQ_Item_18 |  | 146 |  | **0.12** |  | 0.14 |  |
| BCAQ_Item_10 |  | - |  | BCAQ_Item_19 |  | 147 |  | 0.54 | *** | < .001 |  |
| BCAQ_Item_10 |  | - |  | BCAQ_Item_20 |  | 144 |  | 0.40 | *** | < .001 |  |
| BCAQ_Item_10 |  | - |  | BCAQ_Item_21 |  | 139 |  | 0.36 | *** | < .001 |  |
| BCAQ_Item_10 |  | - |  | BCAQ_Item_22 |  | 141 |  | 0.39 | *** | < .001 |  |
| BCAQ_Item_10 |  | - |  | BCAQ_Item_23 |  | 147 |  | 0.33 | *** | < .001 |  |
| BCAQ_Item_10 |  | - |  | BCAQ_Item_24 |  | 145 |  | 0.46 | *** | < .001 |  |
| BCAQ_Item_10 |  | - |  | BCAQ_Item_25 |  | 147 |  | 0.52 | *** | < .001 |  |
| BCAQ_Item_10 |  | - |  | BCAQ_Item_26 |  | 147 |  | 0.39 | *** | < .001 |  |
| BCAQ_Item_10 |  | - |  | BCAQ_Item_27 |  | 147 |  | 0.48 | *** | < .001 |  |
| BCAQ_Item_11 |  | - |  | BCAQ_Item_12 |  | 147 |  | 0.49 | *** | < .001 |  |
| BCAQ_Item_11 |  | - |  | BCAQ_Item_13 |  | 147 |  | 0.34 | *** | < .001 |  |
| BCAQ_Item_11 |  | - |  | BCAQ_Item_14 |  | 147 |  | 0.34 | *** | < .001 |  |
| BCAQ_Item_11 |  | - |  | BCAQ_Item_15 |  | 147 |  | 0.51 | *** | < .001 |  |
| BCAQ_Item_11 |  | - |  | BCAQ_Item_16 |  | 147 |  | 0.25 | ** | 2.54×10^-3^ |  |
| BCAQ_Item_11 |  | - |  | BCAQ_Item_17 |  | 147 |  | 0.63 | *** | < .001 |  |
| BCAQ_Item_11 |  | - |  | BCAQ_Item_18 |  | 146 |  | **0.11** |  | 0.17 |  |
| BCAQ_Item_11 |  | - |  | BCAQ_Item_19 |  | 147 |  | 0.38 | *** | < .001 |  |
| BCAQ_Item_11 |  | - |  | BCAQ_Item_20 |  | 144 |  | 0.22 | ** | 8.41×10^-3^ |  |
| BCAQ_Item_11 |  | - |  | BCAQ_Item_21 |  | 139 |  | 0.18 | * | 0.03 |  |
| BCAQ_Item_11 |  | - |  | BCAQ_Item_22 |  | 141 |  | 0.21 | * | 0.01 |  |
| BCAQ_Item_11 |  | - |  | BCAQ_Item_23 |  | 147 |  | 0.32 | *** | < .001 |  |
| BCAQ_Item_11 |  | - |  | BCAQ_Item_24 |  | 145 |  | 0.24 | ** | 4.21×10^-3^ |  |
| BCAQ_Item_11 |  | - |  | BCAQ_Item_25 |  | 147 |  | 0.43 | *** | < .001 |  |
| BCAQ_Item_11 |  | - |  | BCAQ_Item_26 |  | 147 |  | 0.43 | *** | < .001 |  |
| BCAQ_Item_11 |  | - |  | BCAQ_Item_27 |  | 147 |  | 0.32 | *** | < .001 |  |
| BCAQ_Item_12 |  | - |  | BCAQ_Item_13 |  | 147 |  | 0.45 | *** | < .001 |  |
| BCAQ_Item_12 |  | - |  | BCAQ_Item_14 |  | 147 |  | 0.42 | *** | < .001 |  |
| BCAQ_Item_12 |  | - |  | BCAQ_Item_15 |  | 147 |  | 0.54 | *** | < .001 |  |
| BCAQ_Item_12 |  | - |  | BCAQ_Item_16 |  | 147 |  | 0.31 | *** | < .001 |  |
| BCAQ_Item_12 |  | - |  | BCAQ_Item_17 |  | 147 |  | 0.60 | *** | < .001 |  |
| BCAQ_Item_12 |  | - |  | BCAQ_Item_18 |  | 146 |  | **0.10** |  | 0.21 |  |
| BCAQ_Item_12 |  | - |  | BCAQ_Item_19 |  | 147 |  | 0.50 | *** | < .001 |  |
| BCAQ_Item_12 |  | - |  | BCAQ_Item_20 |  | 144 |  | **0.15** |  | 0.07 |  |
| BCAQ_Item_12 |  | - |  | BCAQ_Item_21 |  | 139 |  | 0.31 | *** | < .001 |  |
| BCAQ_Item_12 |  | - |  | BCAQ_Item_22 |  | 141 |  | **0.15** |  | 0.08 |  |
| BCAQ_Item_12 |  | - |  | BCAQ_Item_23 |  | 147 |  | 0.54 | *** | < .001 |  |
| BCAQ_Item_12 |  | - |  | BCAQ_Item_24 |  | 145 |  | 0.32 | *** | < .001 |  |
| BCAQ_Item_12 |  | - |  | BCAQ_Item_25 |  | 147 |  | 0.56 | *** | < .001 |  |
| BCAQ_Item_12 |  | - |  | BCAQ_Item_26 |  | 147 |  | 0.48 | *** | < .001 |  |
| BCAQ_Item_12 |  | - |  | BCAQ_Item_27 |  | 147 |  | 0.47 | *** | < .001 |  |
| BCAQ_Item_13 |  | - |  | BCAQ_Item_14 |  | 147 |  | 0.73 | *** | < .001 |  |
| BCAQ_Item_13 |  | - |  | BCAQ_Item_15 |  | 147 |  | 0.40 | *** | < .001 |  |
| BCAQ_Item_13 |  | - |  | BCAQ_Item_16 |  | 147 |  | 0.49 | *** | < .001 |  |
| BCAQ_Item_13 |  | - |  | BCAQ_Item_17 |  | 147 |  | 0.37 | *** | < .001 |  |
| BCAQ_Item_13 |  | - |  | BCAQ_Item_18 |  | 146 |  | **0.07** |  | 0.37 |  |
| BCAQ_Item_13 |  | - |  | BCAQ_Item_19 |  | 147 |  | 0.65 | *** | < .001 |  |
| BCAQ_Item_13 |  | - |  | BCAQ_Item_20 |  | 144 |  | 0.39 | *** | < .001 |  |
| BCAQ_Item_13 |  | - |  | BCAQ_Item_21 |  | 139 |  | 0.30 | *** | < .001 |  |
| BCAQ_Item_13 |  | - |  | BCAQ_Item_22 |  | 141 |  | 0.41 | *** | < .001 |  |
| BCAQ_Item_13 |  | - |  | BCAQ_Item_23 |  | 147 |  | 0.36 | *** | < .001 |  |
| BCAQ_Item_13 |  | - |  | BCAQ_Item_24 |  | 145 |  | 0.45 | *** | < .001 |  |
| BCAQ_Item_13 |  | - |  | BCAQ_Item_25 |  | 147 |  | 0.59 | *** | < .001 |  |
| BCAQ_Item_13 |  | - |  | BCAQ_Item_26 |  | 147 |  | 0.36 | *** | < .001 |  |
| BCAQ_Item_13 |  | - |  | BCAQ_Item_27 |  | 147 |  | 0.76 | *** | < .001 |  |
| BCAQ_Item_14 |  | - |  | BCAQ_Item_15 |  | 147 |  | 0.33 | *** | < .001 |  |
| BCAQ_Item_14 |  | - |  | BCAQ_Item_16 |  | 147 |  | 0.57 | *** | < .001 |  |
| BCAQ_Item_14 |  | - |  | BCAQ_Item_17 |  | 147 |  | 0.38 | *** | < .001 |  |
| BCAQ_Item_14 |  | - |  | BCAQ_Item_18 |  | 146 |  | **0.08** |  | 0.36 |  |
| BCAQ_Item_14 |  | - |  | BCAQ_Item_19 |  | 147 |  | 0.67 | *** | < .001 |  |
| BCAQ_Item_14 |  | - |  | BCAQ_Item_20 |  | 144 |  | 0.45 | *** | < .001 |  |
| BCAQ_Item_14 |  | - |  | BCAQ_Item_21 |  | 139 |  | 0.22 | * | 0.01 |  |
| BCAQ_Item_14 |  | - |  | BCAQ_Item_22 |  | 141 |  | 0.43 | *** | < .001 |  |
| BCAQ_Item_14 |  | - |  | BCAQ_Item_23 |  | 147 |  | 0.38 | *** | < .001 |  |
| BCAQ_Item_14 |  | - |  | BCAQ_Item_24 |  | 145 |  | 0.55 | *** | < .001 |  |
| BCAQ_Item_14 |  | - |  | BCAQ_Item_25 |  | 147 |  | 0.61 | *** | < .001 |  |
| BCAQ_Item_14 |  | - |  | BCAQ_Item_26 |  | 147 |  | 0.40 | *** | < .001 |  |
| BCAQ_Item_14 |  | - |  | BCAQ_Item_27 |  | 147 |  | 0.81 | *** | < .001 |  |
| BCAQ_Item_15 |  | - |  | BCAQ_Item_16 |  | 147 |  | 0.26 | ** | 1.72×10^-3^ |  |
| BCAQ_Item_15 |  | - |  | BCAQ_Item_17 |  | 147 |  | 0.60 | *** | < .001 |  |
| BCAQ_Item_15 |  | - |  | BCAQ_Item_18 |  | 146 |  | **0.15** |  | 0.07 |  |
| BCAQ_Item_15 |  | - |  | BCAQ_Item_19 |  | 147 |  | 0.38 | *** | < .001 |  |
| BCAQ_Item_15 |  | - |  | BCAQ_Item_20 |  | 144 |  | **0.15** |  | 0.07 |  |
| BCAQ_Item_15 |  | - |  | BCAQ_Item_21 |  | 139 |  | 0.31 | *** | < .001 |  |
| BCAQ_Item_15 |  | - |  | BCAQ_Item_22 |  | 141 |  | 0.17 | * | 0.04 |  |
| BCAQ_Item_15 |  | - |  | BCAQ_Item_23 |  | 147 |  | 0.28 | *** | < .001 |  |
| BCAQ_Item_15 |  | - |  | BCAQ_Item_24 |  | 145 |  | 0.26 | ** | 1.81×10^-3^ |  |
| BCAQ_Item_15 |  | - |  | BCAQ_Item_25 |  | 147 |  | 0.47 | *** | < .001 |  |
| BCAQ_Item_15 |  | - |  | BCAQ_Item_26 |  | 147 |  | 0.38 | *** | < .001 |  |
| BCAQ_Item_15 |  | - |  | BCAQ_Item_27 |  | 147 |  | 0.29 | *** | < .001 |  |
| BCAQ_Item_16 |  | - |  | BCAQ_Item_17 |  | 147 |  | 0.31 | *** | < .001 |  |
| BCAQ_Item_16 |  | - |  | BCAQ_Item_18 |  | 146 |  | **0.04** |  | 0.61 |  |
| BCAQ_Item_16 |  | - |  | BCAQ_Item_19 |  | 147 |  | 0.61 | *** | < .001 |  |
| BCAQ_Item_16 |  | - |  | BCAQ_Item_20 |  | 144 |  | 0.34 | *** | < .001 |  |
| BCAQ_Item_16 |  | - |  | BCAQ_Item_21 |  | 139 |  | 0.18 | * | 0.03 |  |
| BCAQ_Item_16 |  | - |  | BCAQ_Item_22 |  | 141 |  | 0.34 | *** | < .001 |  |
| BCAQ_Item_16 |  | - |  | BCAQ_Item_23 |  | 147 |  | 0.32 | *** | < .001 |  |
| BCAQ_Item_16 |  | - |  | BCAQ_Item_24 |  | 145 |  | 0.49 | *** | < .001 |  |
| BCAQ_Item_16 |  | - |  | BCAQ_Item_25 |  | 147 |  | 0.51 | *** | < .001 |  |
| BCAQ_Item_16 |  | - |  | BCAQ_Item_26 |  | 147 |  | 0.23 | ** | 4.33×10^-3^ |  |
| BCAQ_Item_16 |  | - |  | BCAQ_Item_27 |  | 147 |  | 0.57 | *** | < .001 |  |
| BCAQ_Item_17 |  | - |  | BCAQ_Item_18 |  | 146 |  | 0.17 | * | 0.04 |  |
| BCAQ_Item_17 |  | - |  | BCAQ_Item_19 |  | 147 |  | 0.36 | *** | < .001 |  |
| BCAQ_Item_17 |  | - |  | BCAQ_Item_20 |  | 144 |  | 0.25 | ** | 2.45×10^-3^ |  |
| BCAQ_Item_17 |  | - |  | BCAQ_Item_21 |  | 139 |  | 0.33 | *** | < .001 |  |
| BCAQ_Item_17 |  | - |  | BCAQ_Item_22 |  | 141 |  | 0.22 | ** | 9.14×10^-3^ |  |
| BCAQ_Item_17 |  | - |  | BCAQ_Item_23 |  | 147 |  | 0.42 | *** | < .001 |  |
| BCAQ_Item_17 |  | - |  | BCAQ_Item_24 |  | 145 |  | 0.38 | *** | < .001 |  |
| BCAQ_Item_17 |  | - |  | BCAQ_Item_25 |  | 147 |  | 0.51 | *** | < .001 |  |
| BCAQ_Item_17 |  | - |  | BCAQ_Item_26 |  | 147 |  | 0.51 | *** | < .001 |  |
| BCAQ_Item_17 |  | - |  | BCAQ_Item_27 |  | 147 |  | 0.40 | *** | < .001 |  |
| BCAQ_Item_18 |  | - |  | BCAQ_Item_19 |  | 146 |  | **0.09** |  | 0.29 |  |
| BCAQ_Item_18 |  | - |  | BCAQ_Item_20 |  | 143 |  | **0.04** |  | 0.65 |  |
| BCAQ_Item_18 |  | - |  | BCAQ_Item_21 |  | 139 |  | 0.19 | * | 0.03 |  |
| BCAQ_Item_18 |  | - |  | BCAQ_Item_22 |  | 141 |  | **0.03** |  | 0.71 |  |
| BCAQ_Item_18 |  | - |  | BCAQ_Item_23 |  | 146 |  | 0.26 | ** | 1.30×10^-3^ |  |
| BCAQ_Item_18 |  | - |  | BCAQ_Item_24 |  | 144 |  | 0.25 | ** | 2.89×10^-3^ |  |
| BCAQ_Item_18 |  | - |  | BCAQ_Item_25 |  | 146 |  | 0.21 | * | 0.01 |  |
| BCAQ_Item_18 |  | - |  | BCAQ_Item_26 |  | 146 |  | 0.25 | ** | 2.33×10^-3^ |  |
| BCAQ_Item_18 |  | - |  | BCAQ_Item_27 |  | 146 |  | **0.11** |  | 0.19 |  |
| BCAQ_Item_19 |  | - |  | BCAQ_Item_20 |  | 144 |  | 0.36 | *** | < .001 |  |
| BCAQ_Item_19 |  | - |  | BCAQ_Item_21 |  | 139 |  | 0.22 | * | 0.01 |  |
| BCAQ_Item_19 |  | - |  | BCAQ_Item_22 |  | 141 |  | 0.36 | *** | < .001 |  |
| BCAQ_Item_19 |  | - |  | BCAQ_Item_23 |  | 147 |  | 0.32 | *** | < .001 |  |
| BCAQ_Item_19 |  | - |  | BCAQ_Item_24 |  | 145 |  | 0.51 | *** | < .001 |  |
| BCAQ_Item_19 |  | - |  | BCAQ_Item_25 |  | 147 |  | 0.68 | *** | < .001 |  |
| BCAQ_Item_19 |  | - |  | BCAQ_Item_26 |  | 147 |  | 0.40 | *** | < .001 |  |
| BCAQ_Item_19 |  | - |  | BCAQ_Item_27 |  | 147 |  | 0.66 | *** | < .001 |  |
| BCAQ_Item_20 |  | - |  | BCAQ_Item_21 |  | 136 |  | 0.36 | *** | < .001 |  |
| BCAQ_Item_20 |  | - |  | BCAQ_Item_22 |  | 141 |  | 0.65 | *** | < .001 |  |
| BCAQ_Item_20 |  | - |  | BCAQ_Item_23 |  | 144 |  | 0.30 | *** | < .001 |  |
| BCAQ_Item_20 |  | - |  | BCAQ_Item_24 |  | 142 |  | 0.48 | *** | < .001 |  |
| BCAQ_Item_20 |  | - |  | BCAQ_Item_25 |  | 144 |  | 0.33 | *** | < .001 |  |
| BCAQ_Item_20 |  | - |  | BCAQ_Item_26 |  | 144 |  | 0.29 | *** | < .001 |  |
| BCAQ_Item_20 |  | - |  | BCAQ_Item_27 |  | 144 |  | 0.42 | *** | < .001 |  |
| BCAQ_Item_21 |  | - |  | BCAQ_Item_22 |  | 136 |  | 0.34 | *** | < .001 |  |
| BCAQ_Item_21 |  | - |  | BCAQ_Item_23 |  | 139 |  | 0.32 | *** | < .001 |  |
| BCAQ_Item_21 |  | - |  | BCAQ_Item_24 |  | 138 |  | 0.26 | ** | 2.00×10^-3^ |  |
| BCAQ_Item_21 |  | - |  | BCAQ_Item_25 |  | 139 |  | 0.36 | *** | < .001 |  |
| BCAQ_Item_21 |  | - |  | BCAQ_Item_26 |  | 139 |  | 0.38 | *** | < .001 |  |
| BCAQ_Item_21 |  | - |  | BCAQ_Item_27 |  | 139 |  | 0.21 | * | 0.01 |  |
| BCAQ_Item_22 |  | - |  | BCAQ_Item_23 |  | 141 |  | 0.32 | *** | < .001 |  |
| BCAQ_Item_22 |  | - |  | BCAQ_Item_24 |  | 140 |  | 0.39 | *** | < .001 |  |
| BCAQ_Item_22 |  | - |  | BCAQ_Item_25 |  | 141 |  | 0.33 | *** | < .001 |  |
| BCAQ_Item_22 |  | - |  | BCAQ_Item_26 |  | 141 |  | 0.31 | *** | < .001 |  |
| BCAQ_Item_22 |  | - |  | BCAQ_Item_27 |  | 141 |  | 0.41 | *** | < .001 |  |
| BCAQ_Item_23 |  | - |  | BCAQ_Item_24 |  | 145 |  | 0.30 | *** | < .001 |  |
| BCAQ_Item_23 |  | - |  | BCAQ_Item_25 |  | 147 |  | 0.40 | *** | < .001 |  |
| BCAQ_Item_23 |  | - |  | BCAQ_Item_26 |  | 147 |  | 0.48 | *** | < .001 |  |
| BCAQ_Item_23 |  | - |  | BCAQ_Item_27 |  | 147 |  | 0.47 | *** | < .001 |  |
| BCAQ_Item_24 |  | - |  | BCAQ_Item_25 |  | 145 |  | 0.63 | *** | < .001 |  |
| BCAQ_Item_24 |  | - |  | BCAQ_Item_26 |  | 145 |  | 0.43 | *** | < .001 |  |
| BCAQ_Item_24 |  | - |  | BCAQ_Item_27 |  | 145 |  | 0.52 | *** | < .001 |  |
| BCAQ_Item_25 |  | - |  | BCAQ_Item_26 |  | 147 |  | 0.48 | *** | < .001 |  |
| BCAQ_Item_25 |  | - |  | BCAQ_Item_27 |  | 147 |  | 0.61 | *** | < .001 |  |
| BCAQ_Item_26 |  | - |  | BCAQ_Item_27 |  | 147 |  | 0.39 | *** | < .001 |  |
|  | | | | | | | | | | | |
|  | | | | | | | | | | | |

*Note*. * p < .05, ** p < .01, *** p < .001; Non-significant correlations are highlighted in grey to identify items that display no or only a limited correlation with the remaining items.
